# Supplementary material for: Immune-mediated tubule atrophy promotes acute kidney injury to chronic kidney disease transition
Source: Nat Commun. 2022 Aug 19;13:4892. doi: 10.1038/s41467-022-32634-0 (PMC9391331; doi:10.1038/s41467-022-32634-0)
Supplement: Supplementary file 1 — Supplementary Information [file 41467_2022_32634_MOESM1_ESM.pdf]

Supplementary Information

Manuscript: Immune-mediated Tubule Atrophy Promotes Acute Kidney Injury to Chronic Kidney Disease Transition

Leyuan Xu[1,*], Jiankan Guo[1], Dennis G. Moledina[1], Lloyd G. Cantley[1,*]

[1]Department of Internal Medicine/Section of Nephrology, Yale University School of Medicine

Corresponding authors:

Leyuan Xu, Ph.D.

Lloyd G. Cantley, M.D.

Department of Internal Medicine/Section of Nephrology

Yale University School of Medicine

PO Box 208029

New Haven, CT 06520

Email: leyuan.xu@yale.edu or lloyd.cantley@yale.edu

Supplementary Table 1. Two-way ANOVA for gene expression analysis.

	Time Factor	Model Factor	Interaction
Havcr1	p<0.0001	p<0.0001	p=0.0005
Lcn2	p<0.0001	p<0.0001	p<0.0001
Lrp2	p<0.0001	p<0.0001	p=0.0015
Slc34a1	p<0.0001	p=0.0047	p=0.0571
Slc13a3	p<0.0001	p=0.0012	p<0.0001
Cd68	p<0.0001	p<0.0001	p=0.0003
F4/80	p<0.0001	p<0.0001	p<0.0001
Itgax	p<0.0001	p<0.0001	p<0.0001
Ly6g	p<0.0001	p=0.3364	p=0.1749
Cd3e	p<0.0001	p<0.0001	p<0.0001
Cd4	p<0.0001	p<0.0001	p<0.0001
Cd8a	p<0.0001	p<0.0001	p<0.0001
Ccl7	p<0.0001	p=0.0012	p<0.0001
Ccl8	p<0.0001	p=0.0005	p=0.0065
Ccl12	p<0.0001	p<0.0001	p<0.0001
Cxcl16	p<0.0001	p<0.0001	p=0.0002
Ccr1	p<0.0001	p=0.0452	p=0.0055
Ccr2	p<0.0001	p<0.0001	p<0.0001
Cxcr6	p<0.0001	p<0.0001	p<0.0001
Il1b	p<0.0001	p<0.0001	p<0.0001
Tnf	p<0.0001	p<0.0001	p<0.0001
Ltb	p<0.0001	p<0.0001	p=0.0005
Fasl	p<0.0001	p<0.0001	p<0.0001
Cd40lg	p<0.0001	p<0.0001	p<0.0001
H2-Aa	p<0.0001	p<0.0001	p<0.0001
H2-Ab1	p<0.0001	p<0.0001	p<0.0001
Cd74	p<0.0001	p<0.0001	p<0.0001
Arg1	p<0.0001	p=0.2365	p=0.3366
Mrc1	p<0.0001	p<0.0001	p<0.0001
Msr1	p<0.0001	p=0.0001	p=0.0597
Pdgfb	p<0.0001	p<0.0001	p<0.0001
Tgfb1	p<0.0001	p<0.0001	p<0.0001

Supplementary Table 2. The number of cells in each cluster/kidney from single cell-RNA-sequencing analysis.

	CONTROL		IRI/CL-NX Day 7		IRI/CL-NX Day 14		IRI/CL-NX Day 30		U-IRI Day 7		U-IRI Day 14		U-IRI Day 30	
	Rep. 1	Rep. 2	Rep. 1	Rep. 2	Rep. 1	Rep. 2	Rep. 1	Rep. 2	Rep. 1	Rep. 2	Rep. 1	Rep. 2	Rep. 1	Rep. 2
PT-S1	1131	2026	429	984	716	1057	819	1505	246	285	68	212	115	436
PT-S2	3853	3101	379	1003	1075	1585	1584	1755	737	604	188	566	714	1554
PT-S3	1957	4376	545	1553	1339	1782	1774	2656	831	691	165	551	657	1043
Injured PT	44	78	66	122	86	62	83	156	183	193	13	53	71	101
TAL	215	330	64	113	133	137	270	579	66	48	22	54	65	131
DCT/CNT	302	291	88	135	219	138	316	590	91	83	14	54	75	123
CD-PC	56	108	33	14	58	27	76	139	44	65	12	26	24	45
CD-IC	107	188	60	136	139	98	393	557	32	45	13	17	21	116
Endotheliu	240	857	137	43	155	96	190	527	197	211	64	129	161	284
Myofibrobla	73	166	119	61	68	52	43	150	111	128	45	117	95	161
Monocyte	24	70	94	68	35	55	51	85	105	122	108	133	56	123
Infil. Mac	101	324	622	104	277	120	305	369	864	688	361	211	245	350
M1 Mac	20	125	231	75	76	23	80	149	342	304	155	59	168	172
M2 Mac	24	57	459	95	69	32	60	113	340	419	96	74	59	60
Prolif. Mac	48	42	176	54	40	48	71	117	197	203	59	57	74	84
Resid. Mac	25	169	278	51	110	49	161	283	282	360	87	148	164	197
pDC	24	127	126	40	65	51	145	253	247	214	112	188	261	384
cDC1	36	80	36	11	52	28	66	92	91	163	44	76	106	154
Prolif.	4	15	7	7	12	2	11	17	20	29	7	22	20	34
cDC2	21	53	445	102	80	50	110	210	409	493	111	116	137	181
PMN #1	6	25	62	108	90	94	57	57	213	313	565	684	159	154
PMN #2	4	13	64	58	36	78	19	66	138	257	660	884	106	152
Naive T	6	25	4	3	19	22	62	75	38	42	20	84	278	266
Th/Treg	33	135	34	29	94	87	251	511	140	141	164	360	755	938
Tc/NKT	67	145	68	39	92	77	158	102	248	192	140	272	487	553
Prolif. T	4	8	9	4	14	5	15	7	21	10	27	34	12	19
B	59	154	30	25	25	56	80	104	89	96	38	89	68	193
Total	8484	1308	4665	5037	5174	5911	7250	1122	6322	6399	3358	5270	5153	8008

Abbreviation: Rep., Replicate; IRI/CL-NX, unilateral ischemia/reperfusion injury with contralateral nephrectomy; U-IRI, unilateral ischemia/reperfusion injury with contralateral kidney intact.

Supplementary Table 3. Two-way ANOVA for histological assessments in Figure 4b and 4c.

	Cortex/Outer medulla Factor	Model Factor	Interaction
F4/80+ area	p=0.0009	p<0.0001	p=0.0087
CD11c+ area	p=0.6218	p<0.0001	p=0.0993
Ly6G+ area	p=0.1808	p<0.0001	p=0.2041
CD3ε+ area	p=0.6869	p<0.0001	p=0.1346
CD4+ area	p=0.0344	p<0.0001	p=0.0099
CD8α+ area	p=0.5851	p<0.0001	p=0.7548

Supplementary Table 4. Pearson correlation coefficient with two-tailed p value analysis for gene expression in the IRI/CL-NX kidneys.

**IRI/CL-NX**		**MΦ/DC**								**Neutrophil**				**T Cell**							
	Gene	*Cd68*		*F4/80*		*Itgax*		*Ccr2*		*Ly6g*		*Ccr1*		*Cd3e*		*Cd4*		*Cd8*		*Cxcr6*	
**Identity**	*Adgre1*	0.9510	*0.0129*																		
	Itgax	0.9697	*0.0063*	0.9616	*0.0090*																
	Ccr2	0.9729	*0.0053*	0.9963	*0.0003*	0.9680	*0.0068*														
	Ly6g	0.6933	*0.1942*	0.4731	*0.4209*	0.6744	*0.2118*	0.5262	*0.3624*												
	Ccr1	0.7569	*0.1385*	0.8790	*0.0496*	0.7157	*0.1740*	0.8639	*0.0590*	0.0622	*0.9209*										
	Cd3e	0.6311	*0.2535*	0.5623	*0.3238*	0.7557	*0.1395*	0.5688	*0.3170*	0.7968	*0.1065*	0.1066	*0.8646*								
	Cd4	0.8760	*0.0514*	0.8326	*0.0801*	0.9461	*0.0149*	0.8401	*0.0749*	0.7990	*0.1049*	0.4754	*0.4183*	0.9032	*0.0356*						
	Cd8a	0.7042	*0.1843*	0.6417	*0.2431*	0.8137	*0.0938*	0.6504	*0.2347*	0.7825	*0.1177*	0.2110	*0.7333*	0.9850	*0.0022*	0.9124	*0.0307*				
	Cxcr6	0.5441	*0.3431*	0.3657	*0.5450*	0.5949	*0.2899*	0.4048	*0.4990*	0.8789	*0.0497*	-0.0926	*0.8823*	0.8916	*0.0421*	0.7320	*0.1597*	0.9001	*0.0374*		
**Macrophage phenotype marker**	*Arg1*	0.6630	*0.2226*	0.7867	*0.1144*	0.5963	*0.2886*	0.7748	*0.1239*												
	Mrc1	0.8767	*0.0510*	0.9635	*0.0083*	0.8584	*0.0626*	0.9553	*0.0113*												
	Msr1	0.8013	*0.1031*	0.8508	*0.0676*	0.7209	*0.1694*	0.8584	*0.0626*												
	Pdgfb	0.8266	*0.0844*	0.9503	*0.0132*	0.8306	*0.0816*	0.9305	*0.0218*												
	Tgfb1	0.8830	*0.0472*	0.9722	*0.0055*	0.8771	*0.0507*	0.9623	*0.0087*												
**Chemokine**	*Ccl2*	0.9800	*0.0034*	0.9196	*0.0270*	0.9450	*0.0153*	0.9461	*0.0149*	0.6816	*0.2051*	0.7210	*0.1693*	0.6306	*0.2540*	0.8330	*0.0799*	0.7263	*0.1646*	0.6054	*0.2793*
	Ccl7	0.9633	*0.0084*	0.9875	*0.0017*	0.9689	*0.0066*	0.9884	*0.0015*	0.5536	*0.3330*	0.8380	*0.0764*	0.5936	*0.2913*	0.8727	*0.0535*	0.6518	*0.2333*	0.3901	*0.5162*
	Ccl8	0.9291	*0.0224*	0.8419	*0.0736*	0.9517	*0.0126*	0.8644	*0.0587*	0.8615	*0.0606*	0.5027	*0.3880*	0.8479	*0.0695*	0.9799	*0.0034*	0.8705	*0.0548*	0.7452	*0.1483*
	Ccl12	0.9295	*0.0222*	0.9836	*0.0025*	0.9438	*0.0158*	0.9765	*0.0043*	0.4731	*0.4210*	0.8670	*0.0570*	0.5450	*0.3421*	0.8401	*0.0749*	0.5994	*0.2854*	0.3030	*0.6202*
	Cxcl16	0.9526	*0.0123*	0.9504	*0.0132*	0.9924	*0.0008*	0.9532	*0.0121*	0.6783	*0.2081*	0.6980	*0.1900*	0.7638	*0.1328*	0.9616	*0.0090*	0.8032	*0.1016*	0.5669	*0.3190*
**Cytokine**	*Tnf*	0.9508	*0.0130*	0.9730	*0.0053*	0.9822	*0.0028*	0.9712	*0.0058*	0.6002	*0.2845*	0.7729	*0.1254*	0.6836	*0.2032*	0.9233	*0.0252*	0.7280	*0.1632*	0.4621	*0.4333*
	Ltb	0.8635	*0.0593*	0.7787	*0.1207*	0.9194	*0.0271*	0.7968	*0.1065*	0.8721	*0.0539*	0.0391	*0.5156*	0.9240	*0.0249*	0.9896	*0.0013*	0.9327	*0.0208*	0.8125	*0.0947*
	Fasl	0.5205	*0.3686*	0.3865	*0.5204*	0.6136	*0.2710*	0.4113	*0.4915*	0.8237	*0.0865*	-0.0840	*0.8931*	0.9456	*0.0151*	0.7668	*0.1303*	0.9446	*0.0155*	0.9832	*0.0026*
	Cd40lg	0.7947	*0.1082*	0.6222	*0.2624*	0.8002	*0.1039*	0.6657	*0.2200*	0.9508	*0.0130*	0.2152	*0.7282*	0.8707	*0.0547*	0.8690	*0.0558*	0.8984	*0.0383*	0.9331	*0.0206*
	Il1b	0.8925	*0.0416*	0.9122	*0.0308*	0.9396	*0.0177*	0.9071	*0.0335*	0.6194	*0.2651*	0.6891	*0.1981*	0.7009	*0.1873*	0.9310	*0.0215*	0.7110	*0.1782*	0.4389	*0.4597*
**MHC**	*H2-Aa*	0.9055	*0.0344*	0.8361	*0.0777*	0.9534	*0.0120*	0.8523	*0.0666*	0.8399	*0.0750*	0.4781	*0.4153*	0.8935	*0.0411*	0.9929	*0.0007*	0.9150	*0.0294*	0.7714	*0.1266*
	H2-Ab1	0.8899	*0.0431*	0.8510	*0.0675*	0.9472	*0.0145*	0.8581	*0.0628*	0.7830	*0.1173*	0.5229	*0.3659*	0.8526	*0.0664*	0.9925	*0.0008*	0.8562	*0.0640*	0.6610	*0.2245*
	Cd74	0.8463	*0.0707*	0.8038	*0.6461*	0.9267	*0.0235*	0.8095	*0.0969*	0.7992	*0.1047*	0.4334	*0.4659*	0.9194	*0.0271*	0.9983	*<0.0001*	0.9199	*0.0269*	0.7400	*0.1528*
**Two-way ANOVA**		R	*p*	R	*p*	R	*p*	R	*p*	R	*p*	R	*p*	R	*p*	R	*p*	R	*p*	R	*p*

Statistically significant correlations between immune cell markers and indicated gene expression are highlighted in yellow, green and tan for macrophage/DC (yellow), neutrophil (green) and T cell (tan), respectively. Pearson correlation coefficient r with two-tailed p value was determined. Abbreviation: IRI/CL-NX, unilateral ischemia/reperfusion injury with contralateral nephrectomy; MHC, major histocompatibility complex; R, Pearson R; p, p value.

Supplementary Table 5. Pearson correlation coefficient with two-tailed p value analysis for gene expression in the U-IRI kidneys.

**U-IRI**		**MΦ/DC**								**Neutrophil**				**T Cell**							
	Gene	*Cd68*		*F4/80*		*Itgax*		*Ccr2*		*Ly6g*		*Ccr1*		*Cd3e*		*Cd4*		*Cd8*		*Cxcr6*	
**Identity**	*Adgre1*	0.9891	*0.0014*																		
	Itgax	0.9868	*0.0018*	0.9817	*0.0030*																
	Ccr2	0.9751	*0.0047*	0.9861	*0.0020*	0.9604	*0.0094*														
	Ly6g	0.8362	*0.0776*	0.8154	*0.0925*	0.8594	*0.0619*	0.8703	*0.0550*												
	Ccr1	0.6588	*0.2266*	0.7023	*0.1861*	0.5999	*0.2849*	0.7687	*0.1288*	0.8040	*0.1011*										
	Cd3e	0.8118	*0.0952*	0.7942	*0.1086*	0.8777	*0.0504*	0.7123	*0.1771*	0.6587	*0.2267*	0.1719	*0.7822*								
	Cd4	0.8987	*0.0381*	0.8873	*0.0446*	0.9529	*0.0122*	0.8362	*0.0776*	0.7955	*0.1076*	0.3757	*0.5331*	0.9762	*0.0044*						
	Cd8a	0.8325	*0.0802*	0.8175	*0.0910*	0.8999	*0.0374*	0.7472	*0.1467*	0.7135	*0.1759*	0.2356	*0.7028*	0.9966	*0.0002*	0.9887	*0.0014*				
	Cxcr6	0.7654	*0.1315*	0.7429	*0.1503*	0.8307	*0.0815*	0.6462	*0.2388*	0.5759	*0.3095*	0.0746	*0.9051*	0.9936	*0.0006*	0.9467	*0.0146*	0.9812	*0.0031*		
**Macrophage phenotype marker**	*Arg1*	0.3503	*0.5632*	0.3740	*0.5351*	0.2136	*0.7301*	0.4501	*0.4469*												
	Mrc1	0.9684	*0.0067*	0.9882	*0.0015*	0.9416	*0.0168*	0.9848	*0.0023*												
	Msr1	0.7774	*0.1218*	0.7720	*0.1261*	0.6747	*0.2116*	0.8227	*0.0872*												
	Pdgfb	0.6737	*0.2124*	0.7121	*0.1772*	0.5719	*0.3138*	0.7463	*0.1474*												
	Tgfb1	0.9803	*0.0033*	0.9763	*0.0044*	0.9398	*0.0176*	0.9795	*0.0035*												
**Chemokine**	*Ccl2*	0.9203	*0.0267*	0.9285	*0.0227*	0.8559	*0.0642*	0.9459	*0.0150*	0.7147	*0.1749*	0.7825	*0.1177*	0.5339	*0.3540*	0.6630	*0.2225*	0.5592	*0.3271*	0.4781	*0.4153*
	Ccl7	0.6896	*0.1976*	0.6893	*0.1980*	0.6647	*0.2210*	0.7986	*0.1051*	0.8946	*0.0404*	0.9761	*0.0044*	0.2907	*0.6351*	0.4884	*0.4038*	0.3598	*0.5519*	0.1878	*0.7623*
	Ccl8	0.9683	*0.0068*	0.9672	*0.0071*	0.9871	*0.0018*	0.9696	*0.0063*	0.9242	*0.0248*	0.6846	*0.2023*	0.8293	*0.0825*	0.9296	*0.0222*	0.8637	*0.0591*	0.7672	*0.1300*
	Ccl12	0.8796	*0.0492*	0.8769	*0.0509*	0.8632	*0.0594*	0.9417	*0.0168*	0.9522	*0.0125*	0.9139	*0.0299*	0.5518	*0.3349*	0.7174	*0.1725*	0.6068	*0.2779*	0.4637	*0.4315*
	Cxcl16	0.9532	*0.0121*	0.9347	*0.0198*	0.9442	*0.0157*	0.9666	*0.0073*	0.9541	*0.0117*	0.8152	*0.0926*	0.7088	*0.1801*	0.8383	*0.0762*	0.7503	*0.1441*	0.6381	*0.2466*
**Cytokine**	*Tnf*	0.9732	*0.0052*	0.9753	*0.0046*	0.9506	*0.0131*	0.9968	*0.0002*	0.8831	*0.0471*	0.8029	*0.1018*	0.6857	*0.2013*	0.8157	*0.0923*	0.7219	*0.1685*	0.6183	*0.2663*
	Ltb	0.9492	*0.0137*	0.9416	*0.0168*	0.9856	*0.0021*	0.9257	*0.0240*	0.9009	*0.0369*	0.5775	*0.3079*	0.9004	*0.0372*	0.9731	*0.0053*	0.9282	*0.0228*	0.8488	*0.0689*
	Fasl	0.7813	*0.1187*	0.7539	*0.1410*	0.8446	*0.0718*	0.6632	*0.2224*	0.6096	*0.2751*	0.1115	*0.8584*	0.9955	*0.0004*	0.9554	*0.0112*	0.9857	*0.0021*	0.9986	*<0.0001*
	Cd40lg	0.8296	*0.0822*	0.7873	*0.1139*	0.8786	*0.0499*	0.7128	*0.1766*	0.6852	*0.2017*	0.2209	*0.7211*	0.9835	*0.0025*	0.9631	*0.0085*	0.9789	*0.0037*	0.9795	*0.0035*
	Il1b	0.7240	*0.1667*	0.7024	*0.1859*	0.7178	*0.1721*	0.7989	*0.1049*	0.9562	*0.0109*	0.9239	*0.0249*	0.4148	*0.4874*	0.5906	*0.2944*	0.4805	*0.4126*	0.3188	*0.6011*
**MHC**	*H2-Aa*	0.9569	*0.0107*	0.9533	*0.0120*	0.9902	*0.0012*	0.9170	*0.0283*	0.8334	*0.0795*	0.4996	*0.3914*	0.9321	*0.0210*	0.9850	*0.0022*	0.9502	*0.0132*	0.8922	*0.0418*
	H2-Ab1	0.9484	*0.0139*	0.9455	*0.0152*	0.9822	*0.0028*	0.9405	*0.0173*	0.9224	*0.0257*	0.6270	*0.2577*	0.8681	*0.0564*	0.9550	*0.0114*	0.9011	*0.0368*	0.8094	*0.0969*
	Cd74	0.9452	*0.0153*	0.9447	*0.0155*	0.9838	*0.0025*	0.9304	*0.0218*	0.8996	*0.0376*	0.5818	*0.3034*	0.8927	*0.0415*	0.9691	*0.0065*	0.9223	*0.0257*	0.8387	*0.0759*
**Two-way ANOVA**		R	*p*	R	*p*	R	*p*	R	*p*	R	*p*	R	*p*	R	*p*	R	*p*	R	*p*	R	*p*

Statistically significant correlations between immune cell markers and indicated gene expression are highlighted in yellow, green, and tan for macrophage/DC (yellow), neutrophil (green), and T cell (tan), respectively. Pearson correlation coefficient r with two-tailed p value was determined. Abbreviation: U-IRI, unilateral ischemia/reperfusion injury with contralateral kidney intact; MHC, major histocompatibility complex; R, Pearson R; p, p value.

Supplementary Table 6. Pearson correlation coefficient with two-tailed p value analysis for gene expression.

	Model	U-IRI						IRI/CL-NX					
	MHC	*H2-Aa*		*H2-Ab1*		*Cd74*		*H2-Aa*		*H2-Ab1*		*Cd74*	
**Cytokine**	*Tnf*	0.9002	*0.0373*	0.9307	*0.0217*	0.9159	*0.0289*	0.9157	*0.0290*	0.9446	*0.0155*	0.9049	*0.0347*
	Ltb	0.9902	*0.0012*	0.9973	*0.0002*	0.9987	*<0.0001*	0.9951	*0.0004*	0.9742	*0.0050*	0.9881	*0.0016*
	Fasl	0.9042	*0.0351*	0.8281	*0.0833*	0.8546	*0.0651*	0.7869	*0.1143*	0.6921	*0.1953*	0.7810	*0.1189*
	Cd40lg	0.9227	*0.0255*	0.8628	*0.0597*	0.8812	*0.0483*	0.9135	*0.0302*	0.8317	*0.0807*	0.8623	*0.0601*
	Il1b	0.6597	*0.2258*	0.7873	*0.1139*	0.7493	*0.1449*	0.9076	*0.0332*	0.9633	*0.0084*	0.9217	*0.0260*
**Two-way ANOVA**		R	*p*	R	*p*	R	*p*	R	*p*	R	*p*	R	*p*

Statistically significant correlations between major histocompatibility complex (MHC) and inflammatory cytokine expression are highlighted in yellow and green for the U-IRI kidneys and IRI/CL-NX kidneys, respectively. Pearson correlation coefficient r with two-tailed p value was determined. Abbreviation: IRI/CL-NX, unilateral ischemia/reperfusion injury with contralateral nephrectomy; U-IRI, unilateral ischemia/reperfusion injury with contralateral kidney intact; R, Pearson R; p, p value.

Supplementary Table 7. Characteristics of participants included in the study.

**Characteristic**	n (%) or Median (IQR)
**Total N**	10
**Demographics**	
Age, years	58 (27, 64)
Female	5 (50%)
Black race	2 (20%)
**Laboratory Features at biopsy**	#VALUE!
Creatinine, mg/dl	4.0 (2.3, 6.6)
Blood Urea Nitrogen, mg/dl	37 (31, 59)
**eGFR trend, ml/min**	
eGFR before AKI	75.7 (70.1, 92.7)
eGFR at biopsy	15.6 (9.5, 31.0)
eGFR 6-month after	58.0 (41.3, 83.7)
eGFR loss from pre-biopsy to biopsy	-61.3 (-70.7, -36.0)
eGFR gain from biopsy to post-biopsy	33.8 (20.9, 49.1)
**Urine tests**	
Albuminuria, mg/g	54 (19, 117)
Leukocyte esterase	
1+	5 (33%)
2+	1 (56%)
Granular casts	
1-5/LPF	3 (38%)
>5/LPF	5 (63%)

Abbreviation: eGFR, estimated glomerular filtration rate, AKI, acute kidney injury; LPF, low power field.

Supplementary Table 8. Diagnosis, etiology, and clinical scenario of participants included in the study.

Case	Diagnosis	Clinical scenario and presumed etiology
# 0284	ATN	Severe AS, Contrast use (Cath/CT) for TAVR workup
# 0252	ATN	Vancomycin, Hypotension, Sepsis
# 0175	ATN	Cirrhosis; Sepsis; NSAIDs
# 0224	ATN	Hypotension; Diarrhea
# 0222	Myeloma	Myeloma cast nephropathy
# 0101	ATN	Hypotension; NSAID
# 0082	ATN	Cisplatin
# 0022	ATN	Hypotension; Cirrhosis; Large Volume Paracentesis; Sepsis
# 0017	ATN	Hypotension; Cirrhosis; Sepsis; Diuresis
# 0034	Arterionephrosclerosis	Chronic NSAID use

Case number represents de-identified study number. Abbreviation: ATN, acute tubular necrosis; NSAID= non-steroidal anti-inflammatory drugs; AS= aortic stenosis; TAVR= transaortic valve replacement; Cath = cardiac catheterization; CT= Computed tomography.

Supplementary Table 9. Primer sequences used for quantitative PCR.

Gene	Forward	Reverse
Ccl7	CCTGGGAAGCTGTTATCTTCAA	GGTTTCTGTTCAGGCACATTTC
Ccl12	GCTGTGATCTTCAGGACCATAC	TGGAACTCTCAGCCTAGACAT
Ccr1	ATACTCTGGAAACACAGACTCAC	CCCACCACTCCAATGATGAA
Cd40lg	CTGAACTGTGAGGAGATGAGAAG	TGTAGAACGGATGCTGCATTA
Cd74	GGTCAAGTCACCCTGTGAAG	GGTGTGACATCAGGGAACATAA
Cxcl16	TGTCCATTCTTTATCAGGTTCCA	AACTCTTCCCATGACCAGTTC
Cxcr6	CTGGGCTTCTCTTCTGATGC	CGTTTGTTCTCCTGGCTGTTA
Fasl	TGGCCCATTTAACAGGGAAC	CAACCTCTTCTCCTCCATTAGC
H2-Aa	GAAACACTGGGAACCTGAGAT	TTCCAGGGTGTGACTCATAAAG
H2-Ab1	CATCTACAACCGGGAGGAGTA	ACGACATTGGGCTGTTCAAG
Il1b	TGTGAAATGCCACCTTTTGA	TGTCCTCATCCTGGAAGGTC
Ly6g	TTGTGGACTCTCACAGAAGC	GTCTTCACGTTGACAGCATTAC
Ltb	CTCATAGGCGCTTGGATGA	GACGTGGCAGTAGAGGTAATAG
Vcam1	ACTCCCGTCATTGAGGATATTG	GTTGTATTCCTGGGAGAGATGTAG

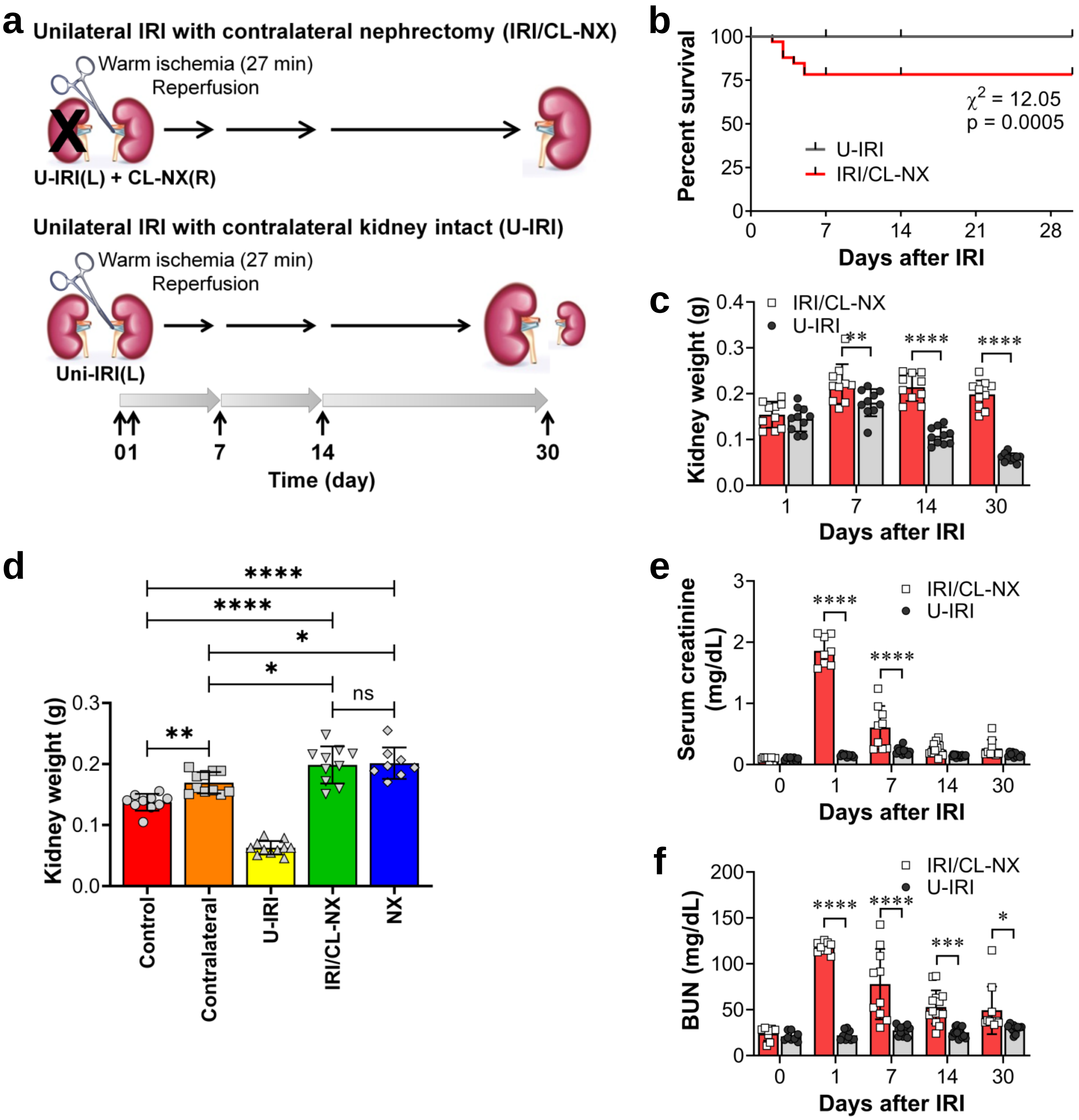

Supplementary Figure 1. Models of ischemia/reperfusion injury. **a.** Scheme of unilateral ischemia/reperfusion injury with contralateral kidney intact (U-IRI) and unilateral ischemia/reperfusion injury with contralateral nephrectomy (IRI/CL-NX) mouse models. Kidneys from mice subjected to 27 minutes of U-IRI were compared to kidneys from mice subjected to 27 minutes of IRI of the left kidney (U-IRI(L)) with contralateral nephrectomy of the right kidney (CL-NX(R)). The mice were sacrificed on day 1, 7, 14 and 30 after injury. **b.** The survival rate was determined in both mouse models. Starting n for U-IRI = 49 mice, and for IRI/CL-NX = 99 mice. Approximately 79% of mice survived for 7 days after IRI/CL-NX; whereas 100% of mice survived after U-IRI. For comparison, Log-rank (Mantel-Cox test) was used to determine Chi square (χ^2) and p value. **c.** Kidney weights were determined on day 1, 7, 14, and 30 after injury. Data are presented as mean ± SD. n=10 kidneys/time point. $p<0.0001$ between models and in time series by two-way ANOVA; **$p<0.01$ and ****$p<0.0001$ in the indicated subgroup analyses by Bonferroni multiple comparison. **d.** Kidney weights were determined on day 30 for U-IRI kidneys and their contralateral kidneys, IRI/CL-NX kidneys, kidneys with CL-NX alone (NX), and age-matched

healthy kidneys (Control). Data are presented as mean ± SD. n = 10 kidneys (8 for NX). $P<0.0001$ among the groups by one-way ANOVA. *$p<0.05$, **$p<0.01$, and ****$p<0.0001$ for the subgroup comparison by Tukey multiple comparison. ns, not statistically significant. **e** and **f**. Serum creatinine and BUN levels of mice at indicated time points after injury. Data are presented as mean ± SD. n=8-14 mice/time point. $p<0.0001$ between models and in time series by two-way ANOVA; *$p<0.05$, ***$p<0.001$, and ****$p<0.0001$ for the subgroup analyses at the indicated time point by Bonferroni multiple comparison. Renal function analyses revealed the expected rise in serum creatinine and BUN on day 1 after injury in the mice subjected to IRI/CL-NX followed by significant improvement on day 7 and return to near baseline by days 14-30 as kidney repair occurred. In contrast, serum creatinine and BUN levels showed minimal changes in mice subjected to the U-IRI alone, reflecting the preserved clearance by the uninjured contralateral kidney.

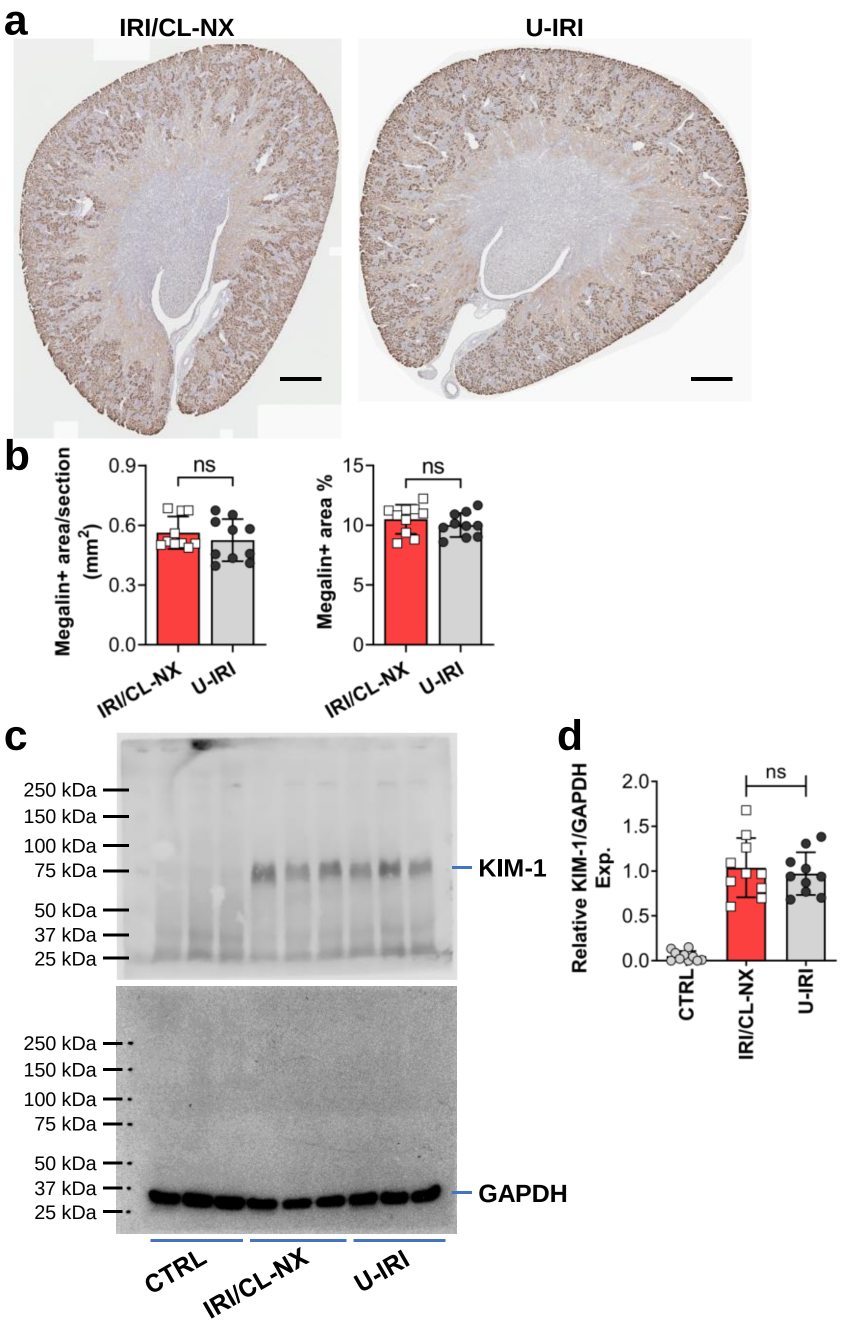

Supplementary Figure 2. Ischemia/reperfusion injury (IRI) leads to equivalent acute kidney injury between IRI with contralateral kidney intact (U-IRI) and unilateral IRI with contralateral nephrectomy (IRI/CL-NX) mouse models. **a.** Midline kidney sections underwent IHC staining for megalin (brown staining) on day 1 after IRI. Scale bars, 0.5 mm. **b.** Megalin-positive area as in (a) was quantified for the entire kidney section (left panel) and as a percentage of the section area (right panel). Data are presented as mean ± SD. n=10 kidneys/group. ns, not statistically significant by unpaired two-tailed t test. (**c**) Western blot analysis for KIM-1 protein expression was performed on whole kidney lysates (each lane is from a separate kidney) on day 1 after IRI and normalized to GAPDH, which was re-probed on the same blot after stripping. The uncropped scans of all blots were supplied in the Source Data files. Due to the limited lanes on one gel, from the same experiment, the remaining samples (7 kidneys/group) were run on another two gels and processed in parallel, in which control, IRI/CL-NX, and U-IRI kidneys were evenly distributed within each gel/blot as shown in the Source Data files (Supplementary Data, Fig.S2). (**d**) Densitometry was analyzed using ImageJ. Data are presented as mean ± SD. n=10 kidneys/group. $p<0.0001$ by two-way ANOVA. ns, not statistically significant at the indicated subgroup comparison by Tukey multiple comparison.

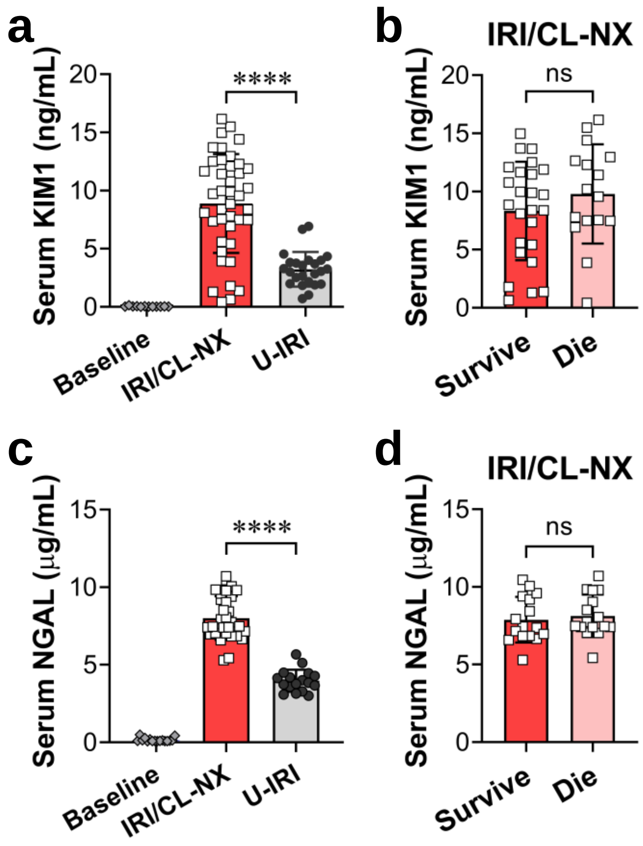

Supplementary Figure 3. Ischemia/reperfusion injury (IRI) leads to acute kidney injury in both models. WT mice were subjected to IRI with contralateral nephrectomy (IRI/CL-NX) or unilateral IRI (U-IRI). **a.** Serum KIM1 levels were determined by ELISA at baseline and on day 1 after injury. Data are presented as mean ± SD. n=12 mice for baseline, n= 41 mice subjected to IRI/CL-NX, and n=24 mice subjected to U-IRI. p<0.0001 by one-way ANOVA; ****p<0.0001 at the indicated subgroup comparison by Tukey multiple comparison. **b.** Serum KIM1 levels in mice subjected to IRI/CL-NX that either survived (and thus were included in the subsequent analyses) or died prior to day 7. Data are presented as mean ± SD. n=25 mice that survived, and n=16 mice that died prior to day 7. ns, not statistically significant by unpaired two-tailed t test. **c.** Serum NGAL levels were determined by ELISA at baseline and on day 1 after injury. Data are presented as mean ± SD. n=16 mice for baseline, n= 32 mice subjected to IRI/CL-NX, and n=17 mice subjected to U-IRI. p<0.0001 by one-way ANOVA; ****p<0.0001 at the indicated subgroup comparison by Tukey multiple comparison. **d.** Serum NGAL levels in mice subjected to IRI/CL-NX that either survived (and thus were included in the subsequent analyses) or died prior to day 7. Data are presented as mean ± SD. n=16 mice that survived, and n=16 mice that died prior to day 7. ns, not statistically significant by unpaired two-tailed t test.

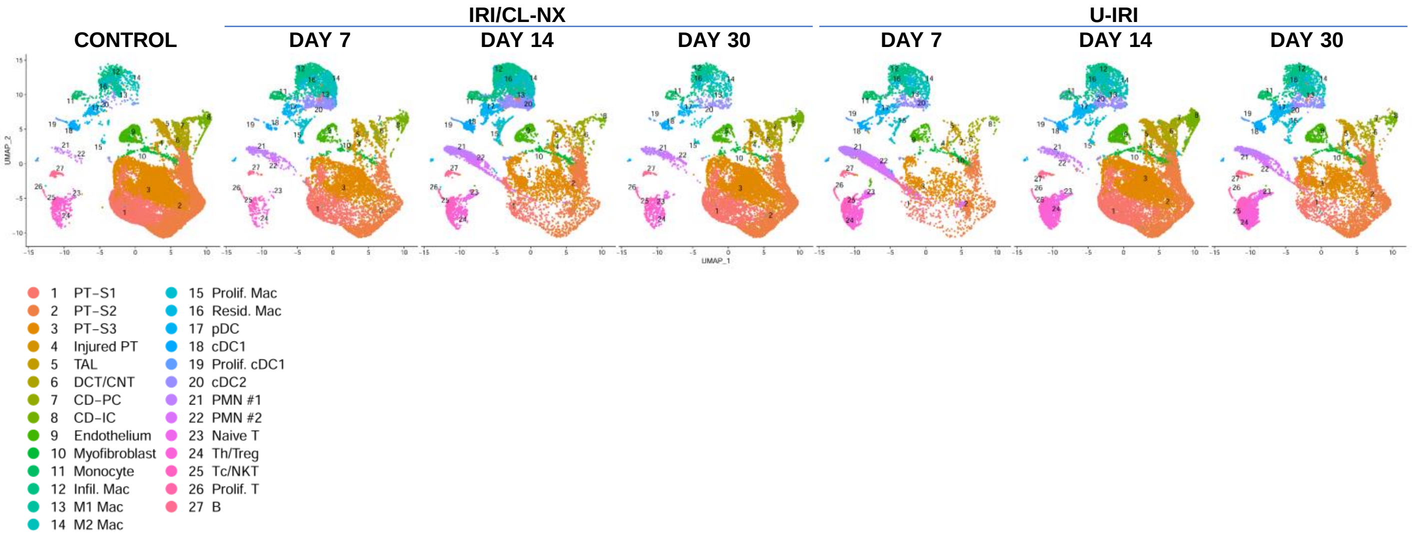

Supplementary Figure 4. UMAP projection of cells from unilateral ischemia/reperfusion injury with contralateral kidney intact (U-IRI), unilateral ischemia/reperfusion injury with contralateral nephrectomy (IRI/CL-NX), and control healthy kidneys. The cell clusters were identified using the composite data from all cells (Figure 3a), and compared in each model at each time point by kidney cell and immune cell lineage-specific marker expression as shown in Figure 3b. Abbreviation: PT, proximal tubule; TAL, thick ascending limb; DCT, distal convoluted tubule; CNT, connecting tubule; CD-PC, collecting duct-principal cell; CD-IC, collecting duct-intercalated cell; Infil. Mac, infiltrating macrophage; Prolif. Mac, proliferating macrophage; Resid. Mac, resident macrophage; pDC, plasmacytoid dendritic cell; cDC, conventional dendritic cell; PMN, polymorphonuclear neutrophils; Th/Treg, T helper/regulatory T cells; Tc/NKT, cytotoxic T/natural killer T cells; B, B cells.

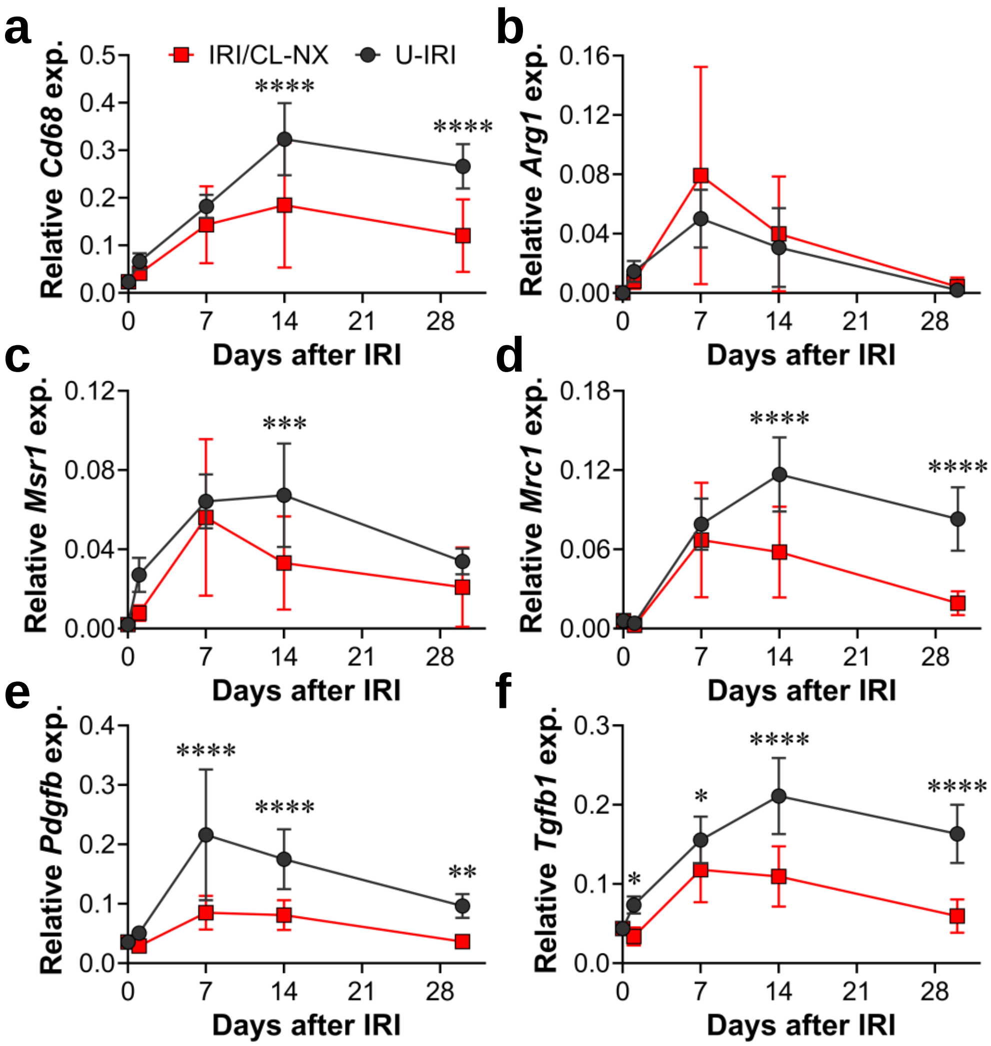

Supplementary Figure 5. Macrophages transition from reparative to profibrotic gene expression in the U-IRI kidney 7 days after injury. Wild-type mice were subjected to 27 minutes of ischemia/reperfusion injury (IRI) with contralateral nephrectomy (IRI/CL-NX) or unilateral IRI (U-IRI) and sacrificed on day 1, 7, 14 and 30 after injury. The injured kidneys and normal control kidneys (defined as day 0) were harvested. **a-f.** Quantitative RT-PCR analysis for indicated genes was performed on whole kidney RNA harvested. Data are presented as mean ± SD. n=10 kidneys/time point. Two-way ANOVA is summarized in Supplementary Table 1. *p<0.05, **p<0.01, ***p<0.001, and ****p<0.0001 at each time point by Bonferroni multiple comparison.

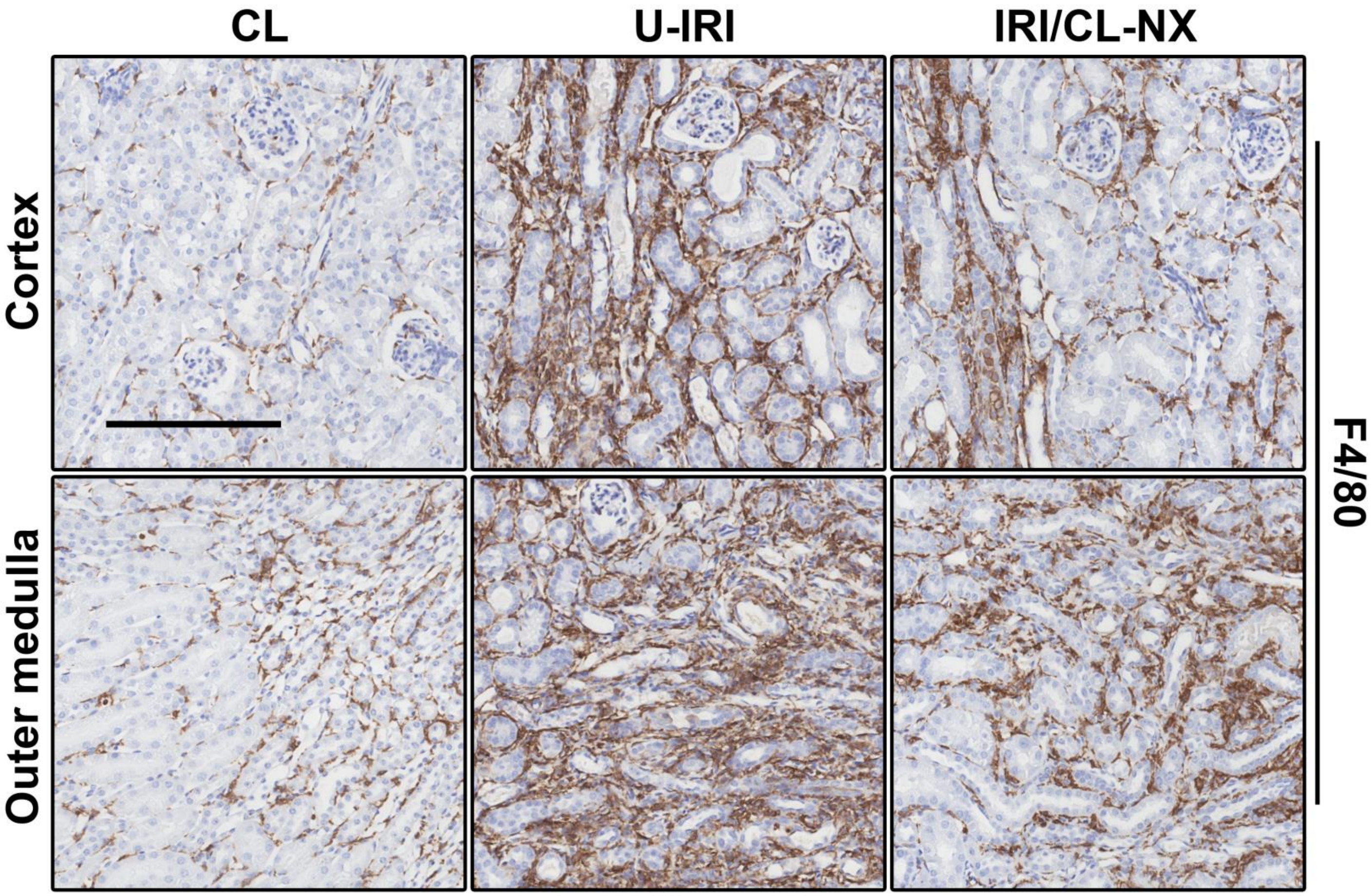

Supplementary Figure 6. Unilateral ischemia/reperfusion injury with contralateral kidney intact (U-IRI) promotes late macrophage accumulation. Contralateral (CL), unilateral IRI (U-IRI) and IRI with contralateral nephrectomy (IRI/CL-NX) kidneys were harvested at 14 days after injury. The kidney sections were immunostained with anti-F4/80. Representative images of kidney sections are shown at 20x magnification. Scale bars, 200 µm. n=10 kidneys/group.

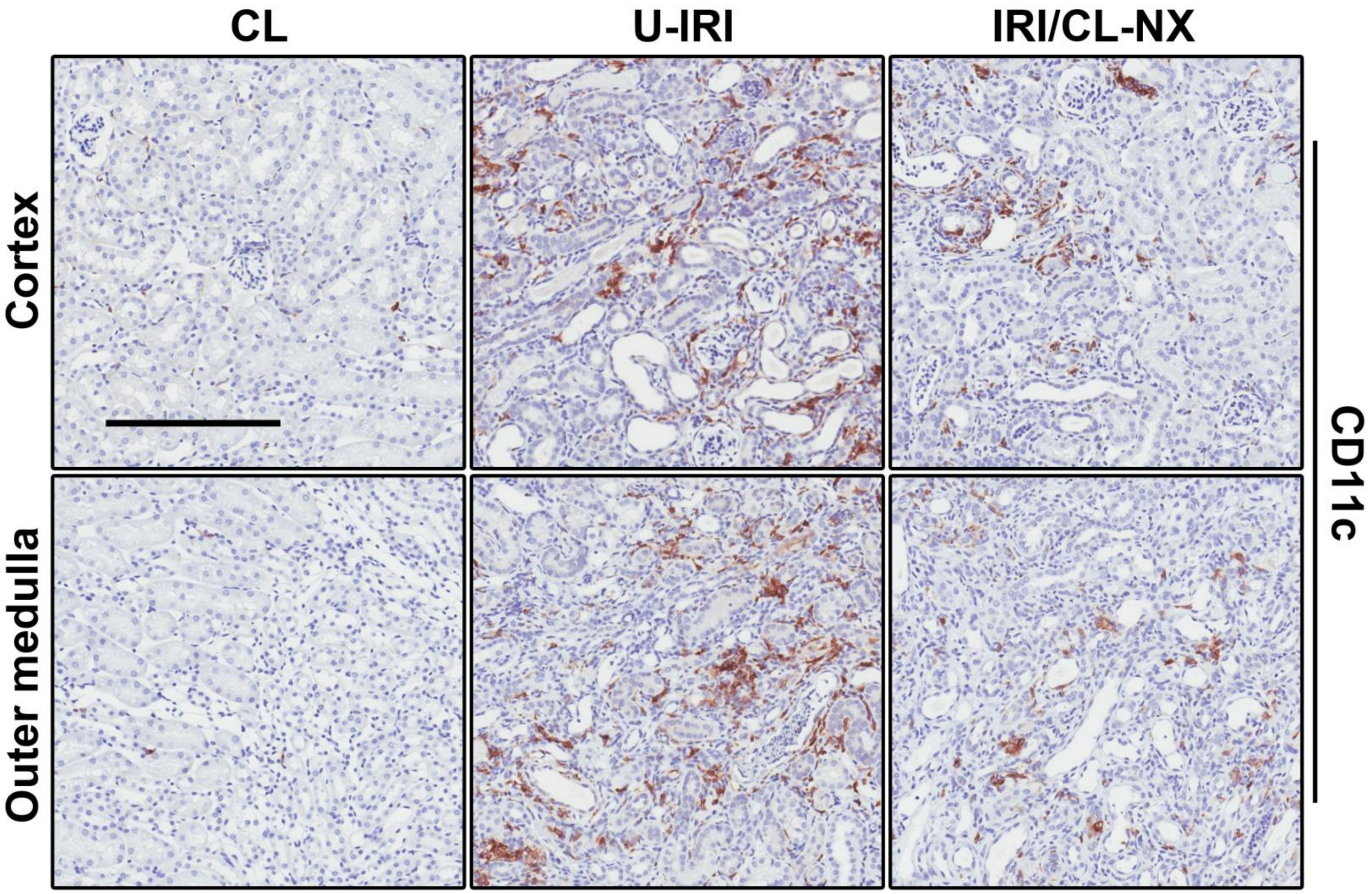

Supplementary Figure 7. Unilateral ischemia/reperfusion injury with contralateral kidney intact (U-IRI) promotes late dendritic cell accumulation. Contralateral (CL), unilateral IRI (U-IRI) and IRI with contralateral nephrectomy (IRI/CL-NX) kidneys were harvested at 14 days after injury. The kidney sections were immunostained with CD11c. Representative images of kidney sections are shown at 20x magnification. Scale bars, 200 µm. n=10 kidneys/group.

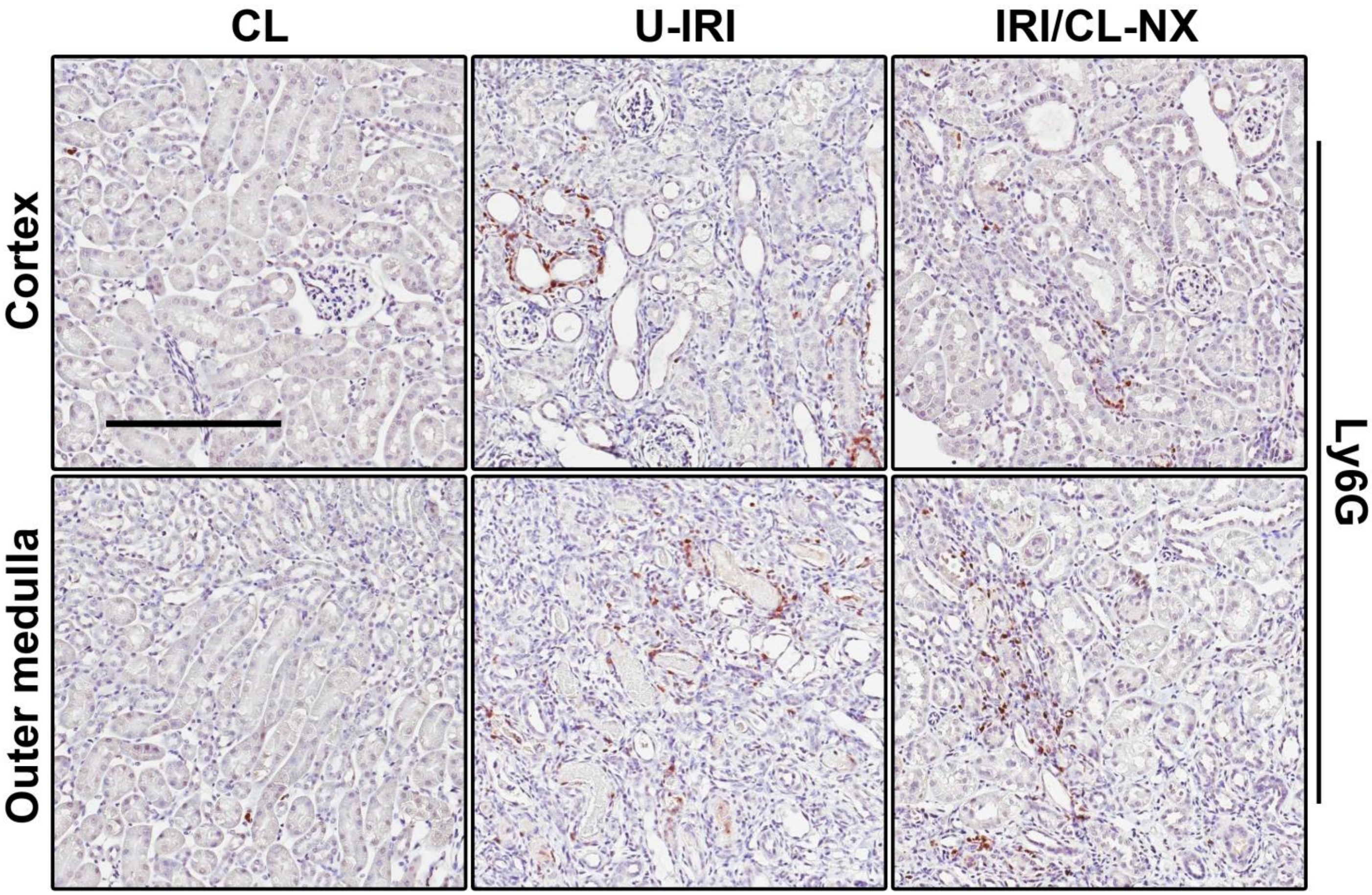

Supplementary Figure 8. Unilateral ischemia/reperfusion injury with contralateral kidney intact (U-IRI) promotes late neutrophil accumulation. Contralateral (CL), unilateral IRI (U-IRI) and IRI with contralateral nephrectomy (IRI/CL-NX) kidneys were harvested at 14 days after injury. The kidney sections were immunostained with Ly6G. Representative images of kidney sections are shown at 20x magnification. Scale bars, 200 µm. n=10 kidneys/group.

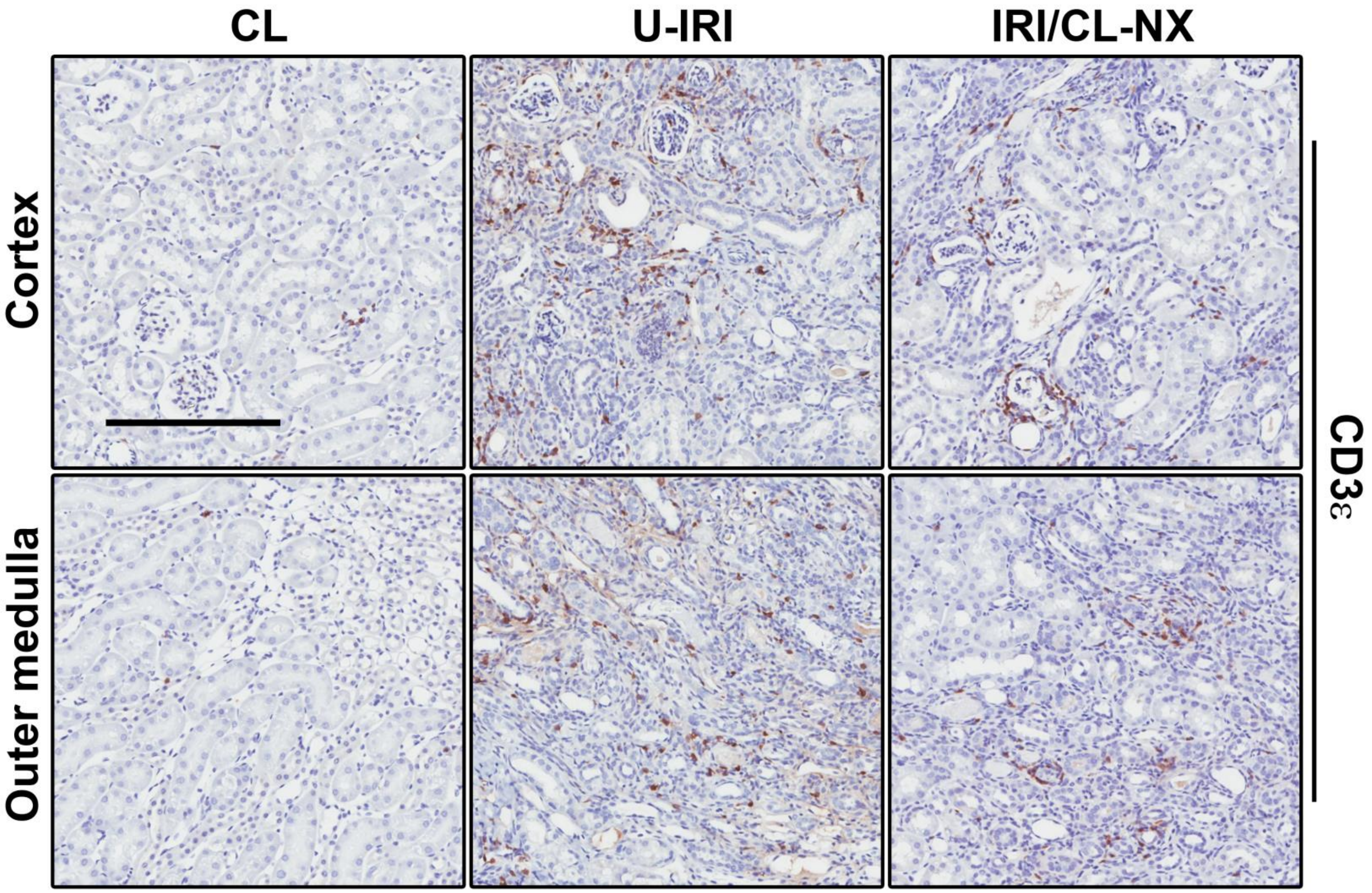

Supplementary Figure 9. Unilateral ischemia/reperfusion injury with contralateral kidney intact (U-IRI) promotes late T cell accumulation. Contralateral (CL), unilateral IRI (U-IRI) and IRI with contralateral nephrectomy (IRI/CL-NX) kidneys were harvested at 14 days after injury. The kidney sections were immunostained with CD3ε. Representative images of kidney sections are shown at 20x magnification. Scale bars, 200 μm. n=10 kidneys/group.

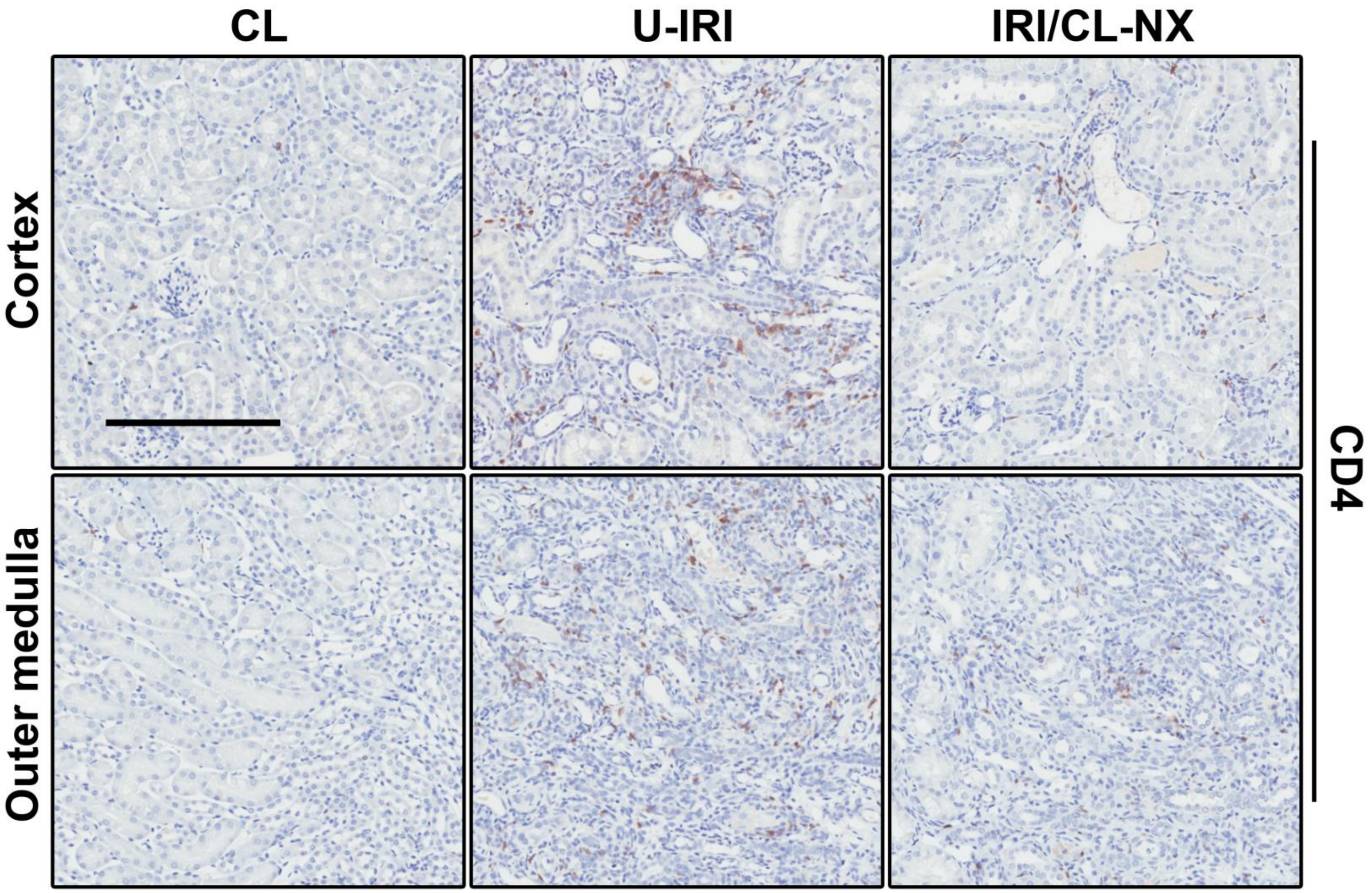

Supplementary Figure 10. Unilateral ischemia/reperfusion injury with contralateral kidney intact (U-IRI) promotes late T helper cell accumulation. Contralateral (CL), unilateral IRI (U-IRI) and IRI with contralateral nephrectomy (IRI/CL-NX) kidneys were harvested at 14 days after injury. The kidney sections were immunostained with CD4. Representative images of kidney sections are shown at 20x magnification. Scale bars, 200 µm. n=10 kidneys/group.

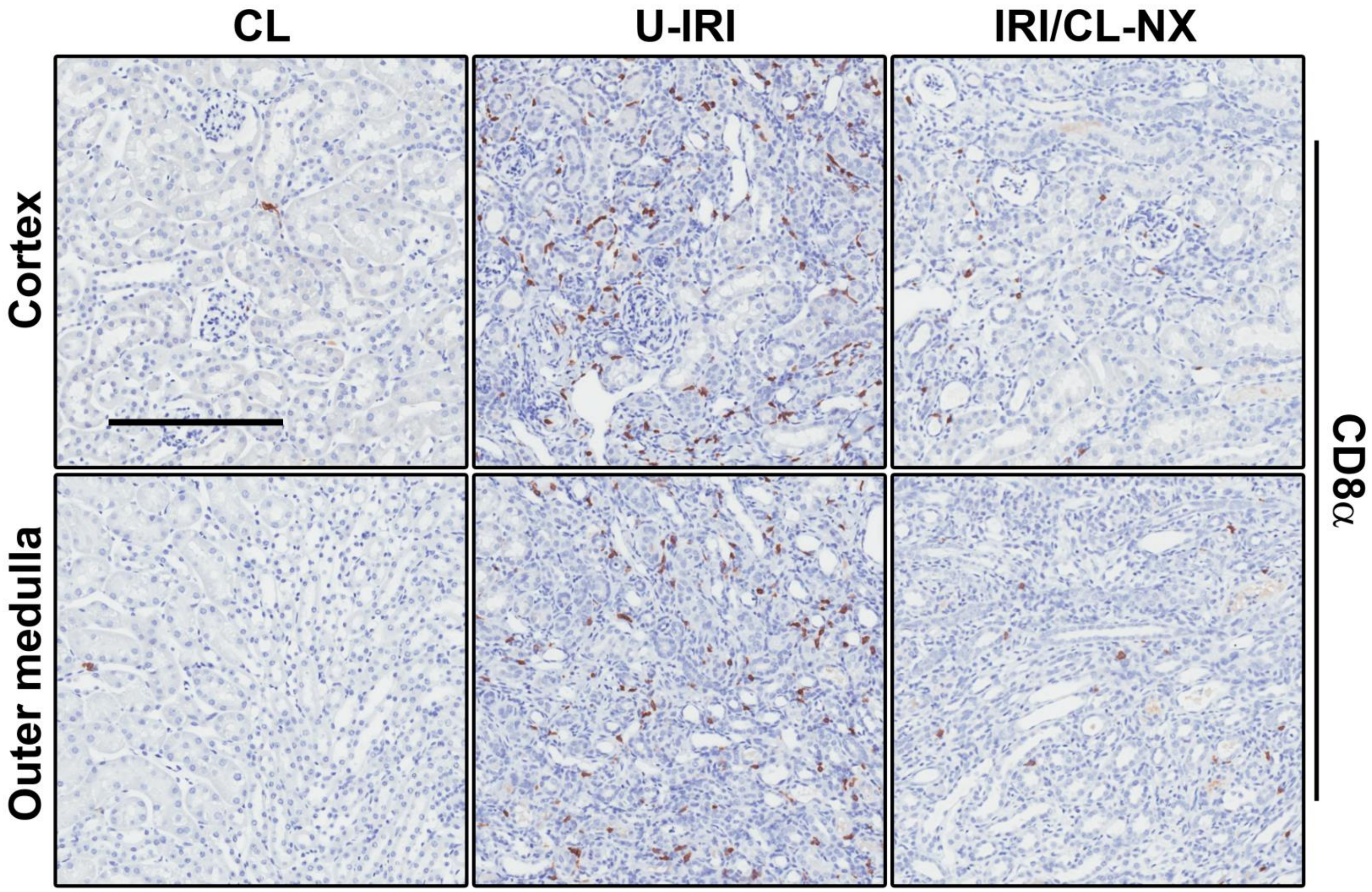

Supplementary Figure 11. Unilateral ischemia/reperfusion injury with contralateral kidney intact (U-IRI) promotes late cytotoxic T cell accumulation. Contralateral (CL), unilateral IRI (U-IRI) and IRI with contralateral nephrectomy (IRI/CL-NX) kidneys were harvested at 14 days after injury. The kidney sections were immunostained with CD8α. Representative images of kidney sections are shown at 20x magnification. Scale bars, 200 µm. n=10 kidneys/group.

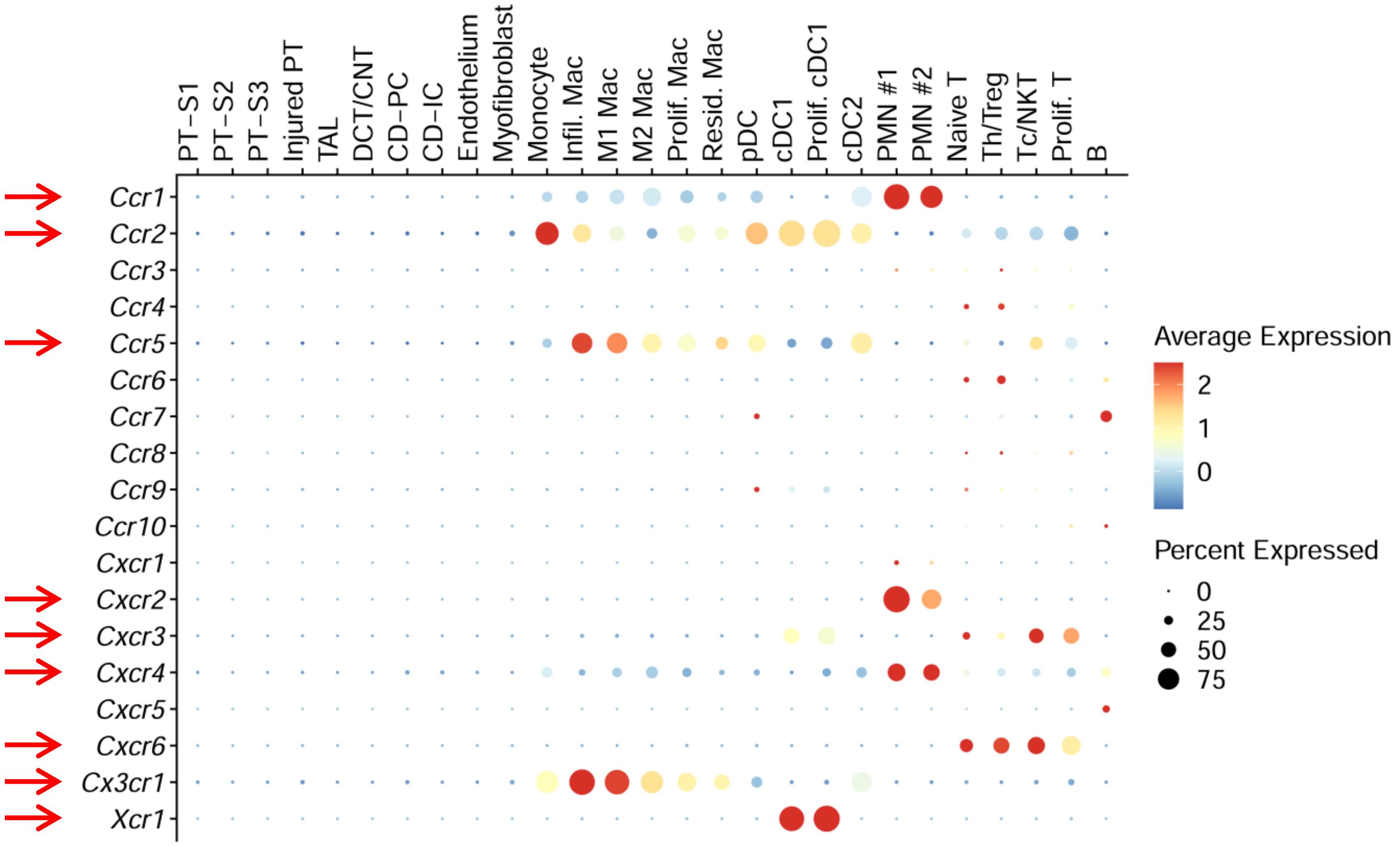

Supplementary Figure 12. The distribution and relative expression of chemokine receptors are shown in a dot plot using the integrated dataset. Abbreviation: PT, proximal tubule; TAL, thick ascending limb; DCT, distal convoluted tubule; CNT, connecting tubule; CD-PC, collecting duct-principal cell; CD-IC, collecting duct-intercalated cell; Infil. Mac, infiltrating macrophage; Prolif. Mac, proliferating macrophage; Resid. Mac, resident macrophage; pDC, plasmacytoid dendritic cell; cDC, conventional dendritic cell; PMN, polymorphonuclear neutrophils; Th/Treg, T helper/regulatory T cells; Tc/NKT, cytotoxic T/natural killer T cells; B, B cells.

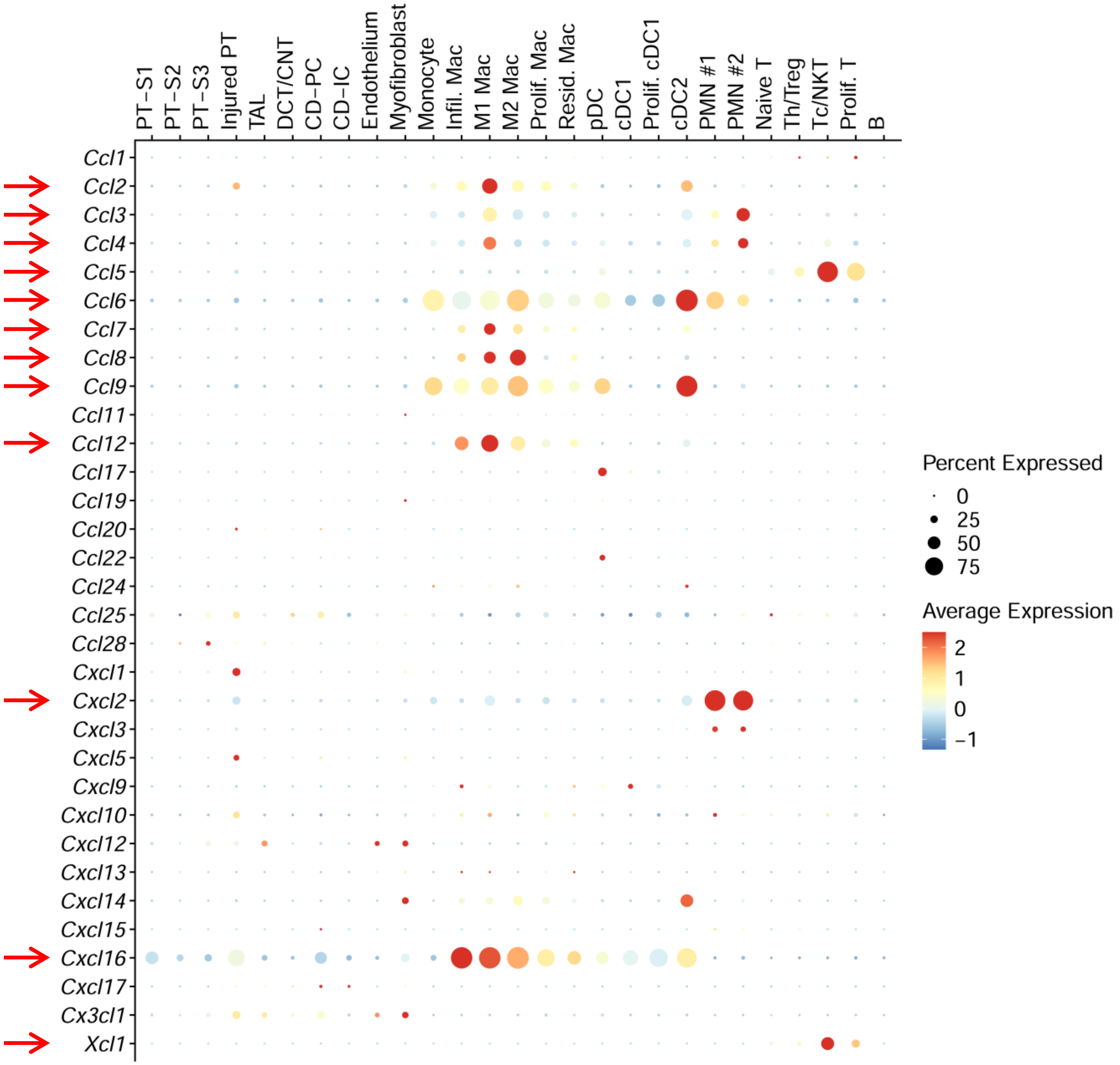

Supplementary Figure 13. The distribution and relative expression of corresponding chemokine ligands are shown in a dot plot using the integrated dataset. Abbreviation: PT, proximal tubule; TAL, thick ascending limb; DCT, distal convoluted tubule; CNT, connecting tubule; CD-PC, collecting duct-principal cell; CD-IC, collecting duct-intercalated cell; Infil. Mac, infiltrating macrophage; Prolif. Mac, proliferating macrophage; Resid. Mac, resident macrophage; pDC, plasmacytoid dendritic cell; cDC, conventional dendritic cell; PMN, polymorphonuclear neutrophils; Th/Treg, T helper/regulatory T cells; Tc/NKT, cytotoxic T/natural killer T cells; B, B cells.

a

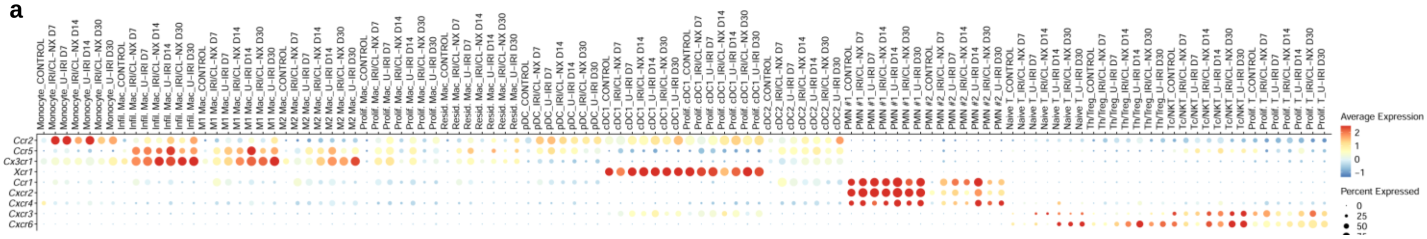

b

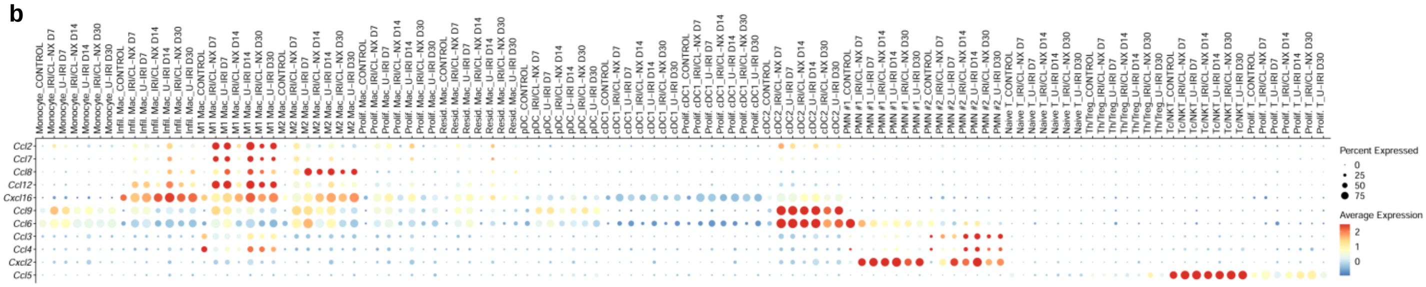

Supplementary Figure 14. The distribution and relative expression of key chemokine receptors (**a**) and corresponding chemokine ligands (**b**) in macrophages, dendritic cells, PMNs, and T cells are shown in dot plots using the split datasets. Abbreviation: PT, proximal tubule; TAL, thick ascending limb; DCT, distal convoluted tubule; CNT, connecting tubule; CD-PC, collecting duct-principal cell; CD-IC, collecting duct-intercalated cell; Infil. Mac, infiltrating macrophage; Prolif. Mac, proliferating macrophage; Resid. Mac, resident macrophage; pDC, plasmacytoid dendritic cell; cDC, conventional dendritic cell; PMN, polymorphonuclear neutrophils; Th/Treg, T helper/regulatory T cells; Tc/NKT, cytotoxic T/natural killer T cells; B, B cells.

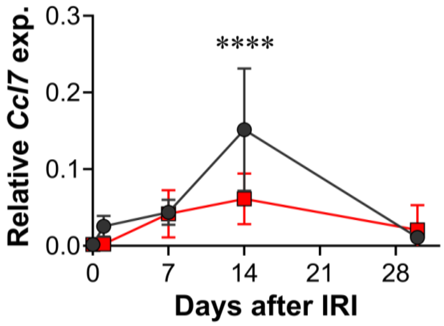

Supplementary Figure 15. Chemokine expression kinetics. Wild-type mice were subjected to 27 minutes of ischemia/reperfusion injury (IRI) with contralateral nephrectomy (IRI/CL-NX) or unilateral IRI (U-IRI). Quantitative RT-PCR analysis for *Ccl7* was performed on whole kidney RNA harvested on day 0 (normal control kidneys), 1, 7, 14, and 30 after injury. Data are presented as mean ± SD. n=10 kidneys/time point. Two-way ANOVA was summarized in Supplemental Table 1. ****p<0.0001 at the indicated time point by Bonferroni multiple comparison.

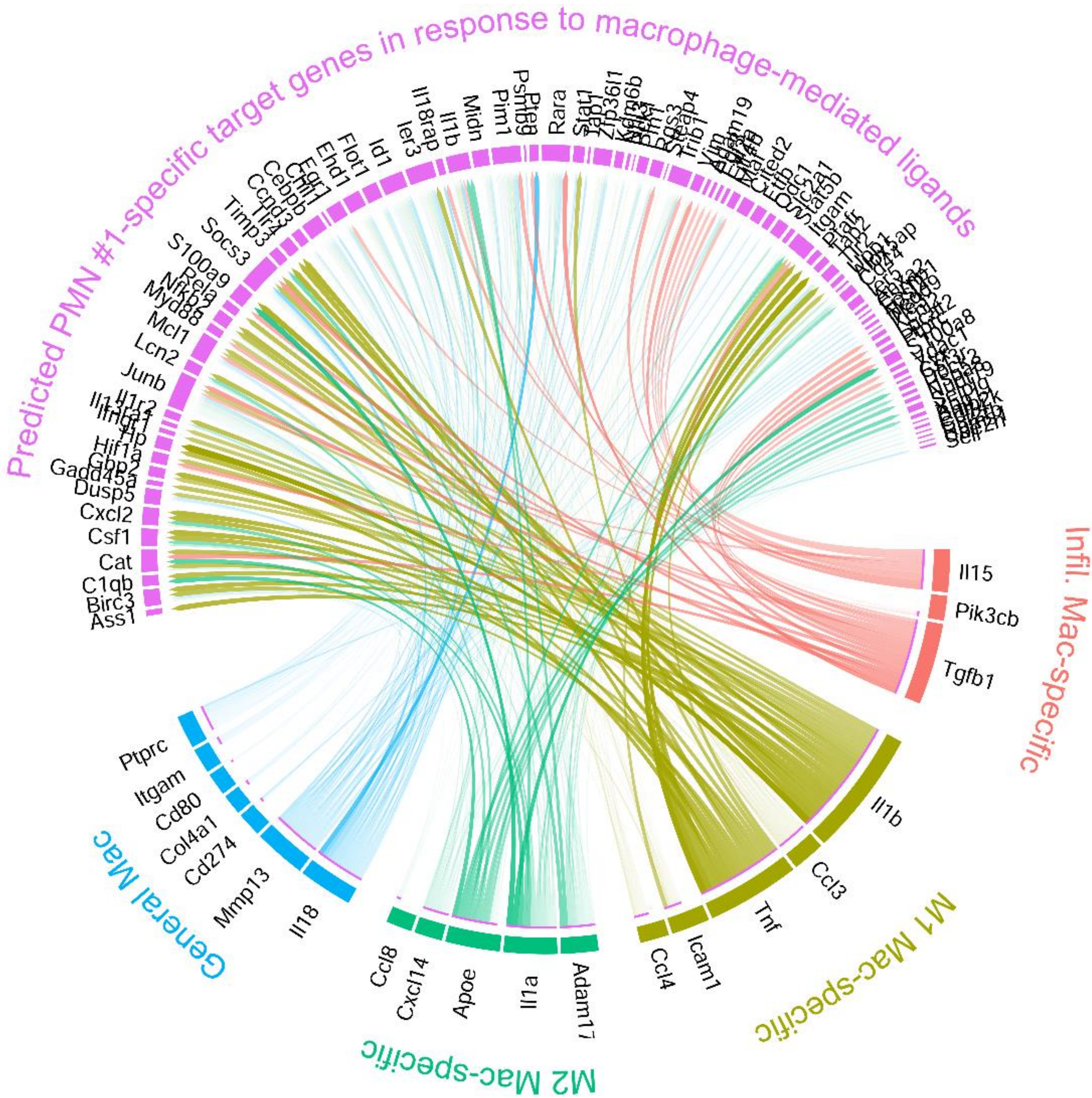

Supplementary Figure 16. Ligand-receptor-target interactions between infiltrating, M1, and M2 macrophages and PMN cluster #1. Based on DEG between U-IRI and IRI/CL-NX kidneys on day 14 after injury, the corresponding ligands that were significantly expressed by the infiltrating, M1, and M2 macrophages were identified and linked to their potential target genes that were upregulated in PMN cluster #1 and visualized using a chord diagram. Abbreviation: Infil. Mac, infiltrating macrophage; PMN, polymorphonuclear neutrophils.

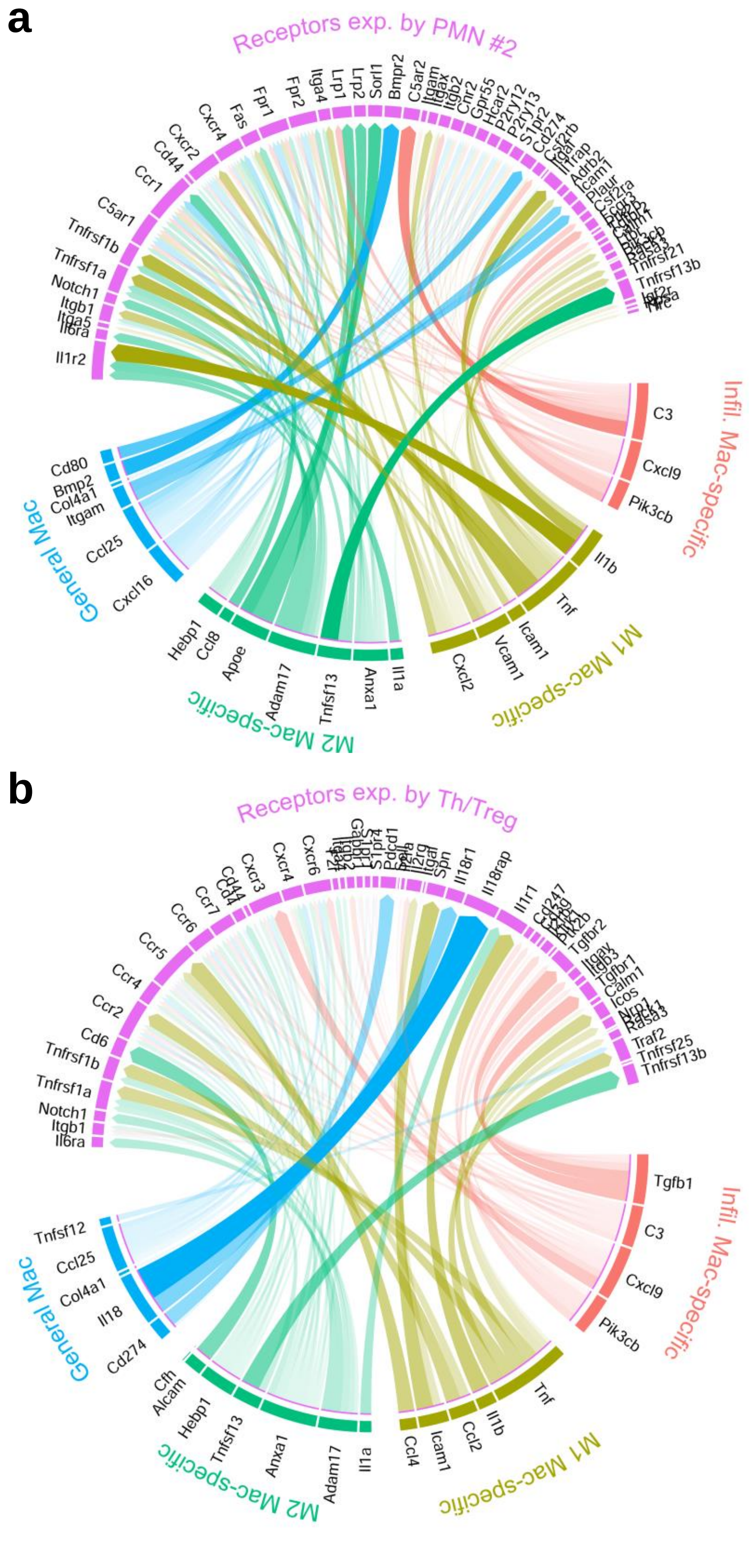

Supplementary Figure 17. Ligand-receptor-target interactions between infiltrating, M1, and M2 macrophages with PMN cluster #2 (**a**) and Cd4+ Th/Treg cells (**b**). Based on DEG between U-IRI and IRI/CL-NX kidneys on day 14 after injury, the corresponding ligands that were significantly expressed by the infiltrating, M1, and M2 macrophages were identified and linked to their corresponding receptors (bottom panel) based on the potential target genes (now shown) for PMN cluster #2 (**a**) and Th/Treg cells (**b**) and visualized using a chord diagram. Abbreviation: Infil. Mac, infiltrating macrophage; PMN, polymorphonuclear neutrophils; Th/Treg, T helper/regulatory T cells.

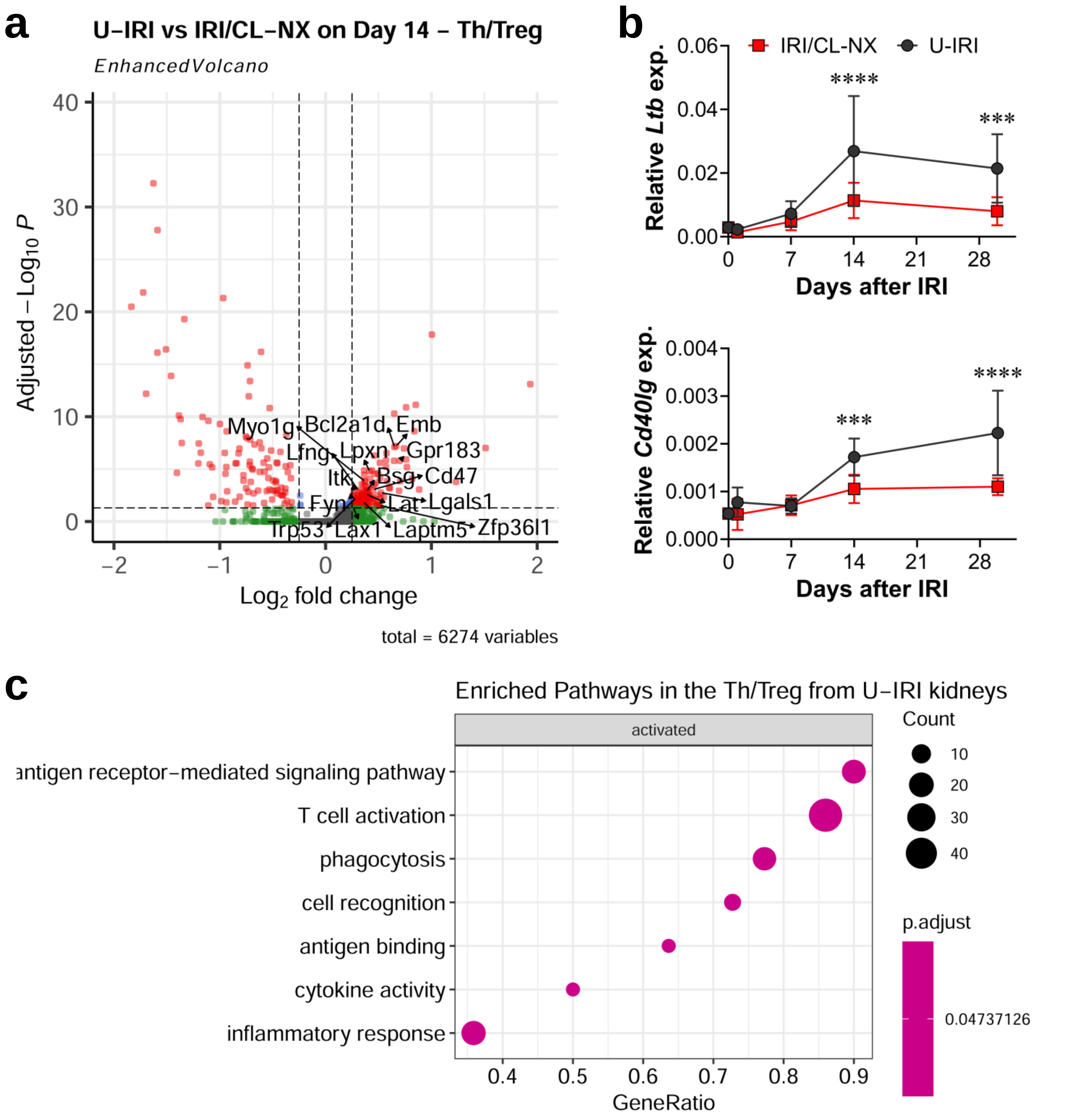

Supplementary Figure 18. U-IRI promotes late CD4+ T cell activation. **a.** Volcano plot demonstrating differential gene expression in U-IRI compared to IRI/CL-NX derived Cd4+ T helper/regulatory T (Th/Treg) cells on day 14 after injury. **b.** Quantitative RT-PCR analysis for indicated genes was performed on whole kidney RNA harvested on day 0, 1, 7, 14, and 30 after injury. Data are presented as mean ± SD. n=10 kidneys/time point/group. Two-way ANOVA summarized in Supplementary Table 1. ***p<0.001, ****p<0.0001 at each time point by Bonferroni multiple comparison. **c.** Based on differentially expressed genes (DEG) between U-IRI and IRI/CL-NX kidneys on day 14 after injury, the top relevant enriched GO terms for Cd4+ Th/Treg cells from U-IRI kidneys are visualized in a dot plot.

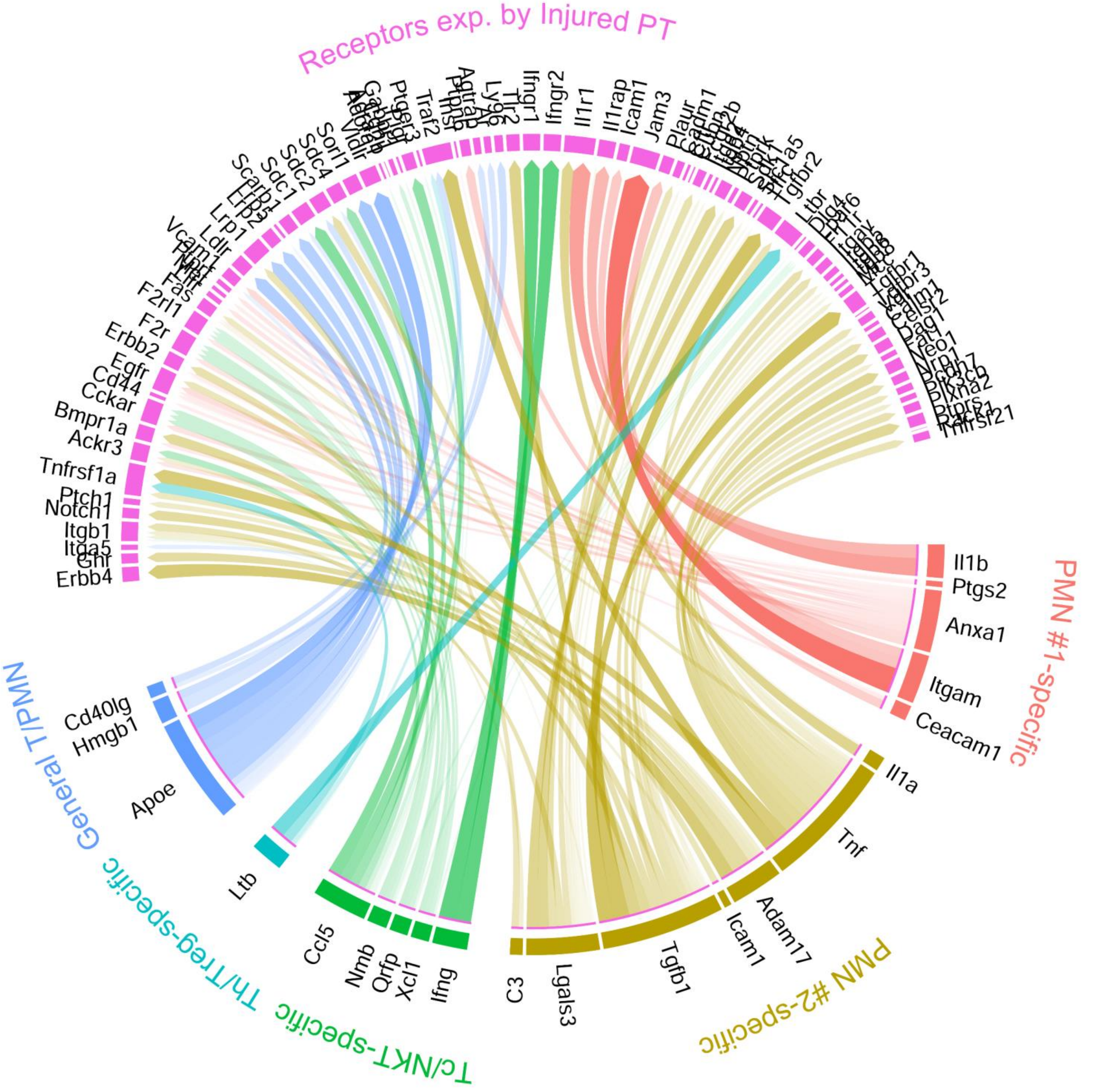

Supplementary Figure 19. Predicted ligand-receptor interactions between PMNs, Cd8a+ Tc/NKT, and Cd4+ Th/Treg cells with injured PT cells. Based on DEG between U-IRI and IRI/CL-NX kidneys on day 14 after injury, the corresponding ligands that were significantly expressed by the PMNs, Cd8a+ Tc/NKT, and Cd4+ Th/Treg cells were identified and linked to their corresponding receptors based on the potential target genes (Figure 7h) for injured PT cells and visualized using a chord diagram. Abbreviation: PT, proximal tubule; PMN, polymorphonuclear neutrophils; Th/Treg, T helper/regulatory T cells; Tc/NKT, cytotoxic T/natural killer T cells.

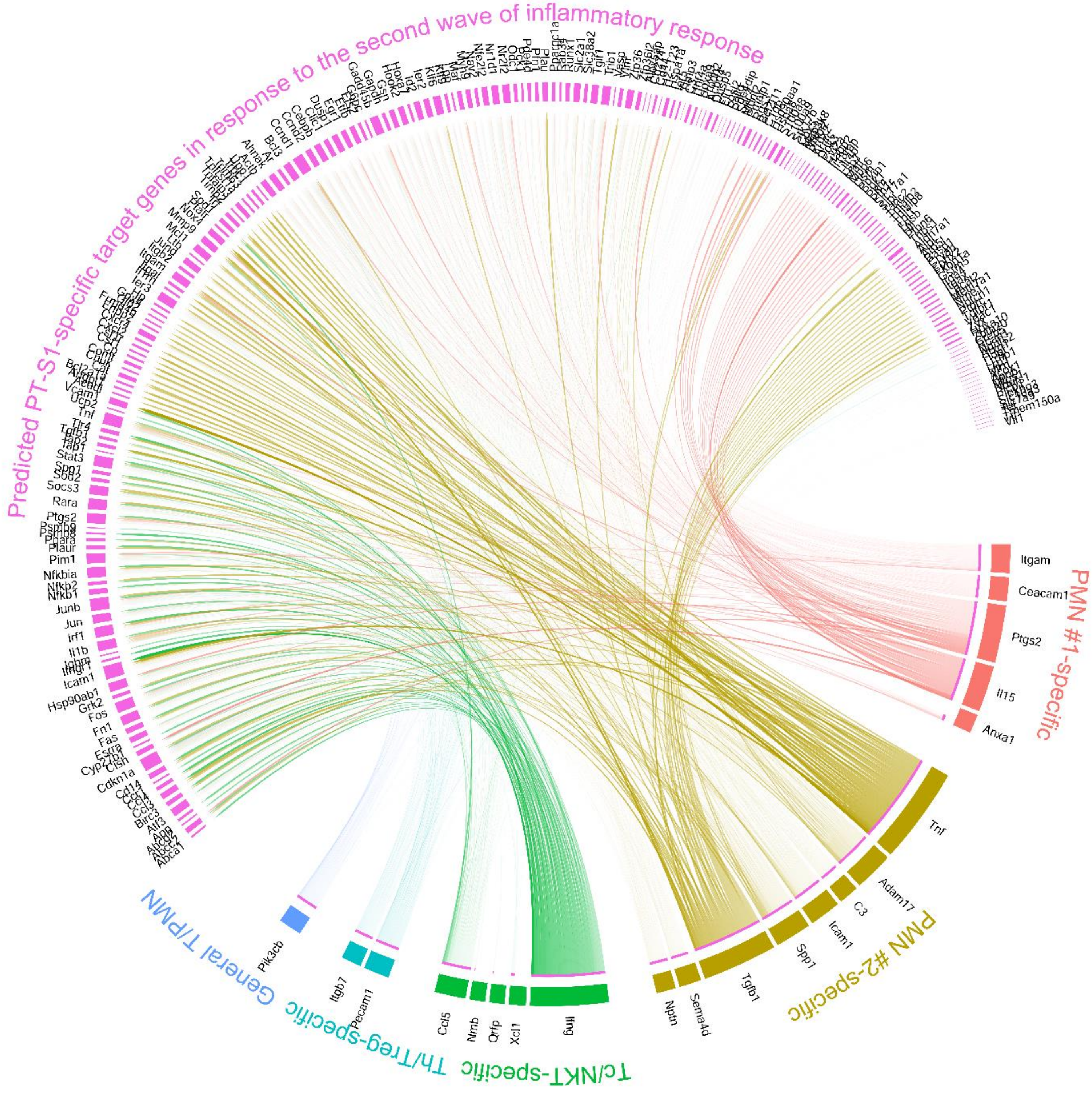

Supplementary Figure 20. Ligand-receptor-target gene expression interactions between PMNs, Cd8a+ Tc/NKT, and Cd4+ Th/Treg cells with proximal tubule cells from segment 1 (PT-S1). Based on DEG between U-IRI and IRI/CL-NX kidneys on day 14 after injury, the corresponding ligands that were significantly expressed by the PMNs, Cd8a+ Tc/NKT, and Cd4+ Th/Treg cells were identified and linked to their potential target genes for PT-S1 cells and visualized using a chord diagram. Abbreviation: PT, proximal tubule; PMN, polymorphonuclear neutrophils; Th/Treg, T helper/regulatory T cells; Tc/NKT, cytooxic T/natural killer T cells.

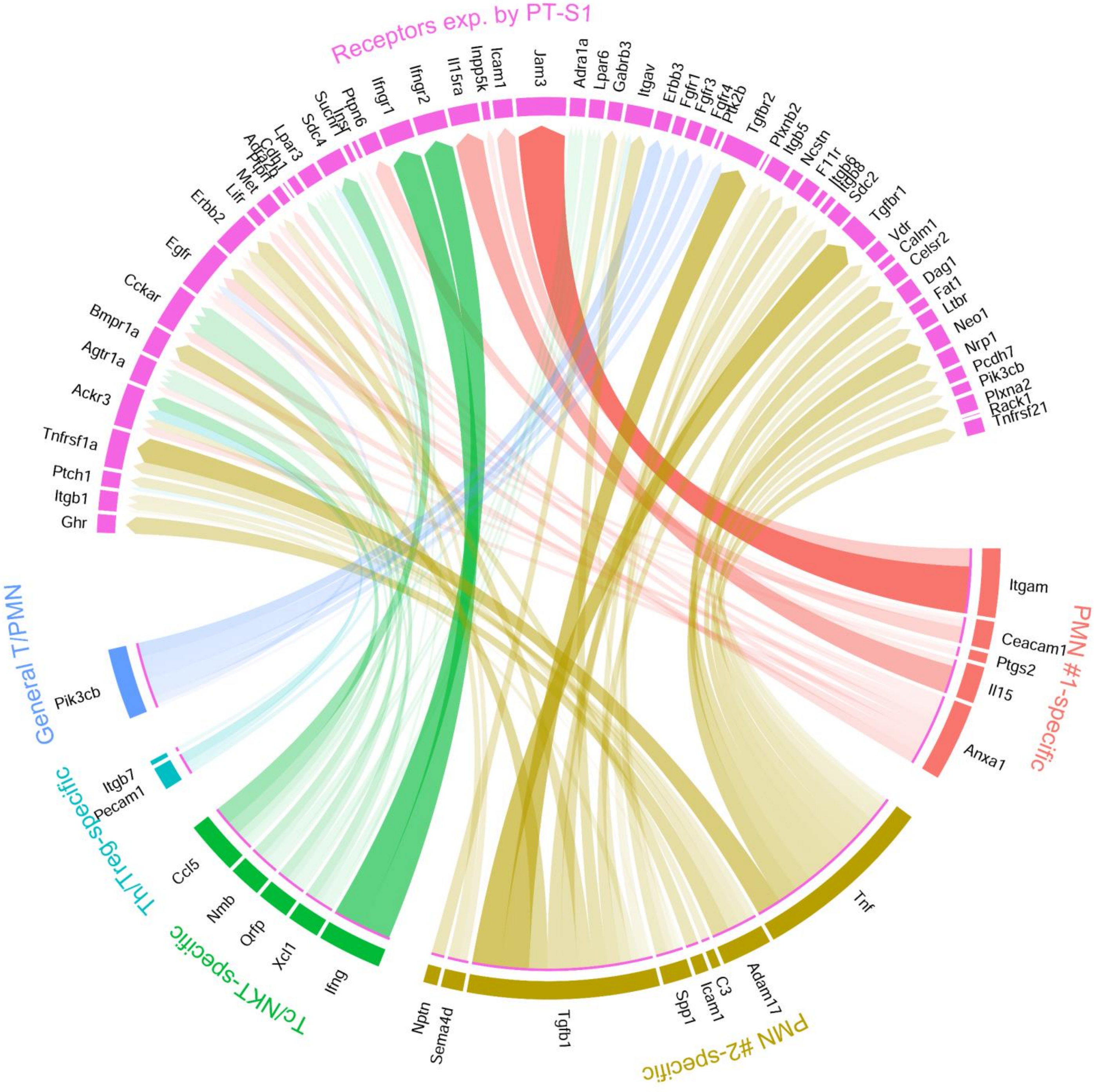

Supplementary Figure 21. Predicted ligand-receptor-target interactions between PMNs, Cd8a+ Tc/NKT, and Cd4+ Th/Treg cells with receptors on proximal tubule cells from segment 1 (PT-S1). Based on DEG between U-IRI and IRI/CL-NX kidneys on day 14 after injury, the corresponding ligands that were significantly expressed by the PMNs, Cd8a+ Tc/NKT, and Cd4+ Th/Treg cells were identified and linked to their corresponding receptors based on the potential target genes (Supplemental Figure 20) for PT-S1 cells and visualized using a chord diagram. Abbreviation: PT, proximal tubule; PMN, polymorphonuclear neutrophils; Th/Treg, T helper/regulatory T cells; Tc/NKT, cytotoxic T/natural killer T cells.

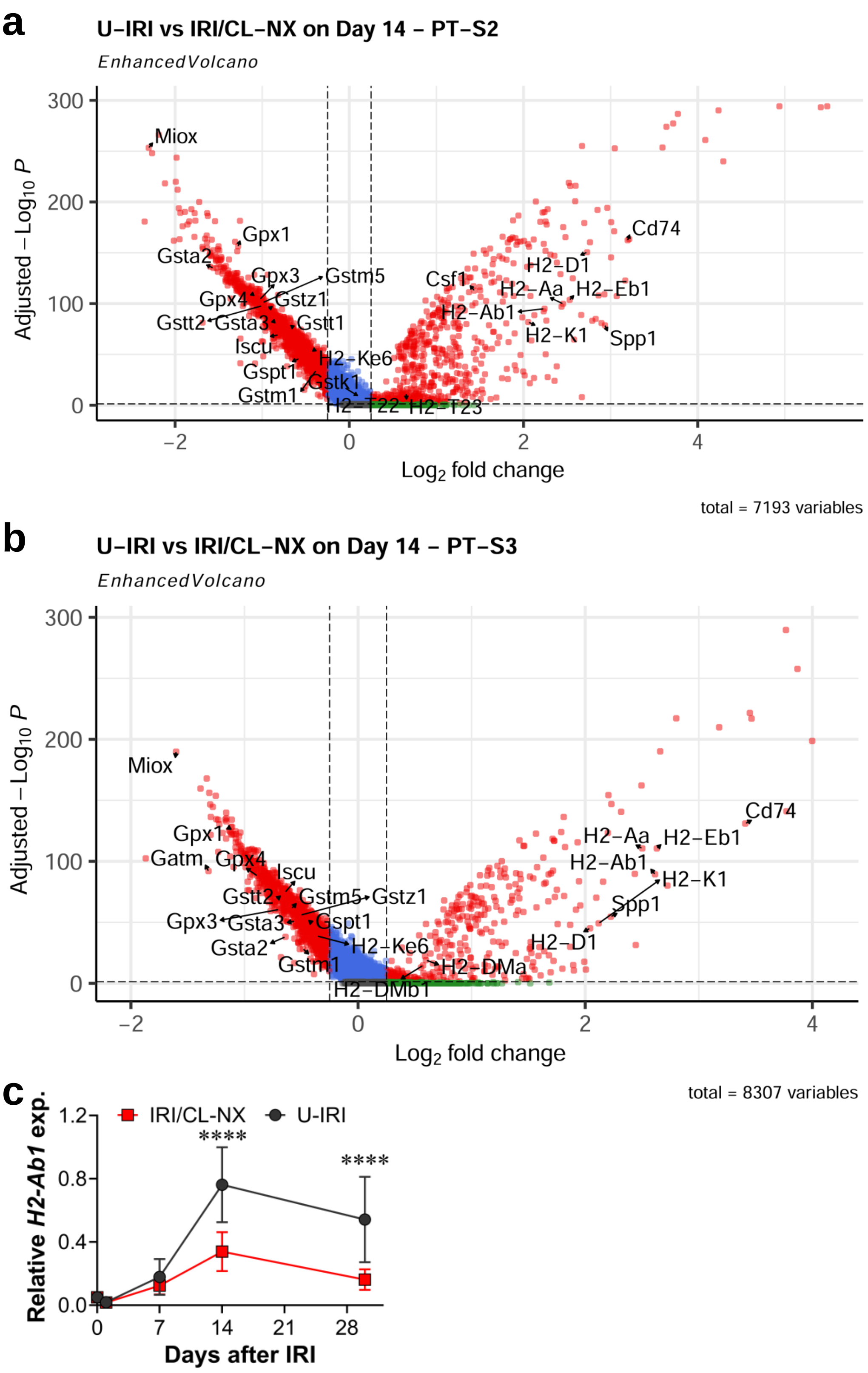

Supplementary Figure 22. Identifying the Injury signature of inflammation-induced tubular stress. **a** and **b.** Volcano plots demonstrating differential gene expression in segment 2 cells from the proximal tubule (PT-S2, **a**) and segment 3 cells from the proximal tubule (PT-S3, **b**) on day 14 after unilateral ischemia/reperfusion injury with contralateral kidney intact (U-IRI) compared to IRI with contralateral nephrectomy (IRI/CL-NX). **c.** Quantitative RT-PCR analysis for *H2-Ab1* was performed on whole kidney RNA harvested on day 0, 1, 7, 14, and 30 after injury. Data are presented as mean ± SD. n=10 kidneys/time point/group. Two-way ANOVA summarized in Supplementary Table 1. ****p<0.0001 at each time point by Bonferroni multiple comparison.

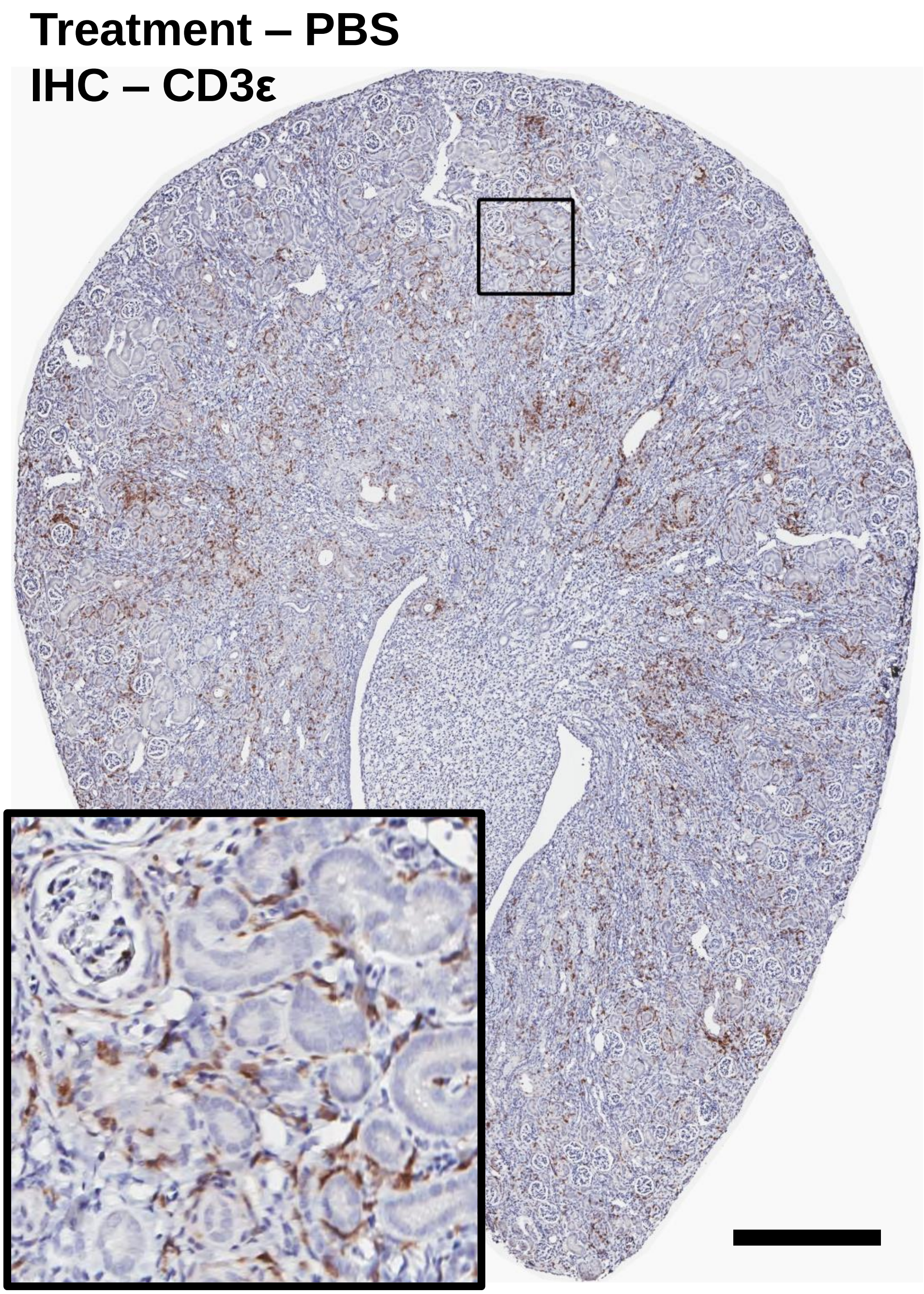

Supplementary Figure 23. Kidney sections from 9 mice treated with PBS on day 30 after unilateral ischemia/reperfusion injury (U-IRI) were immunostained with anti-CD3ε and a representative image is shown with higher magnification insert of the area designated by the box. Scale bar, 400 μm.

Treatment – Ab-Thy1.2 + Ab-Ly6G
IHC – CD3ε

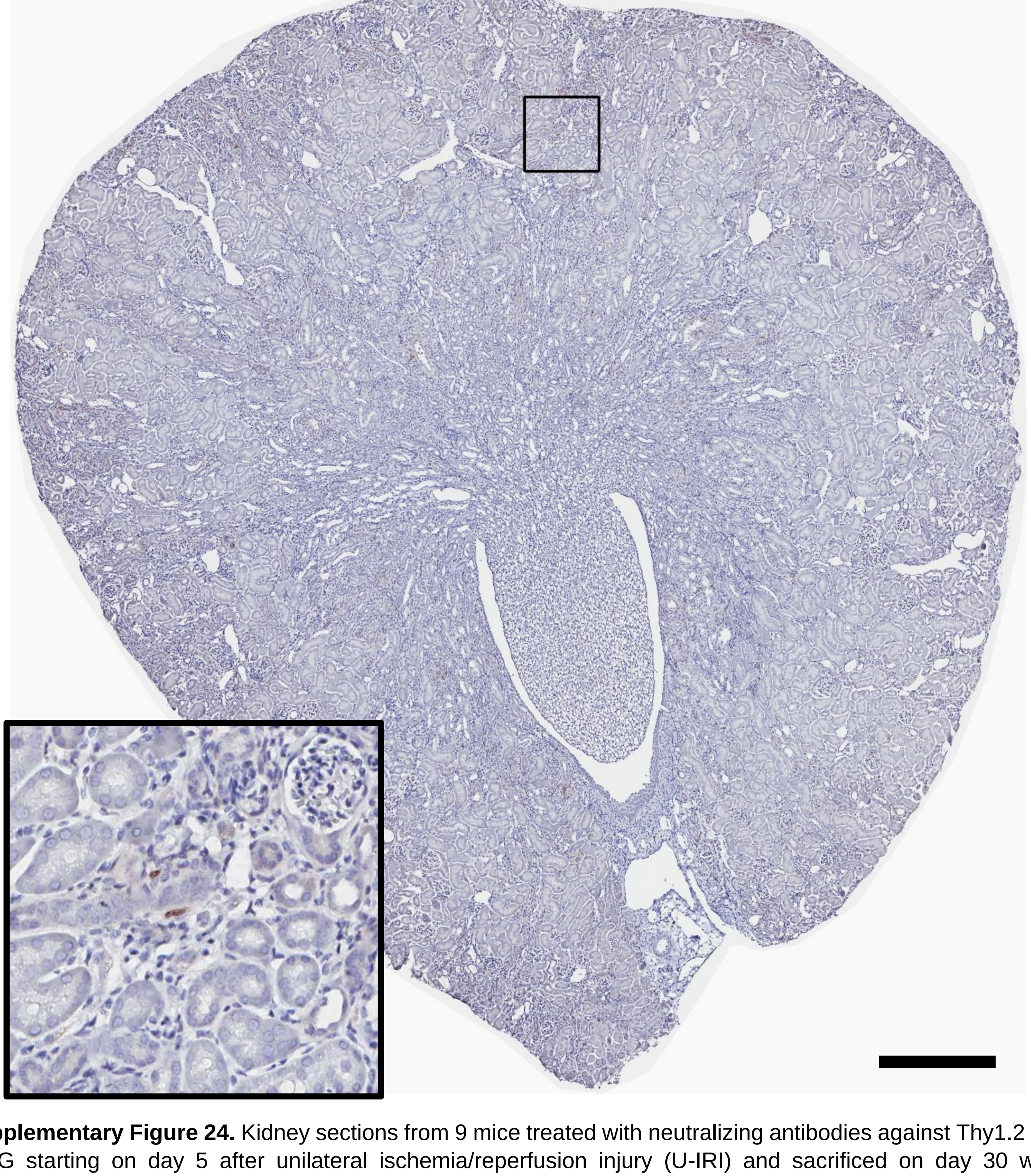

Supplementary Figure 24. Kidney sections from 9 mice treated with neutralizing antibodies against Thy1.2 and Ly6G starting on day 5 after unilateral ischemia/reperfusion injury (U-IRI) and sacrificed on day 30 were immunostained with anti-CD3ε and a representative image is shown with higher magnification insert of the area designated by the box. Scale bar, 400 μm.

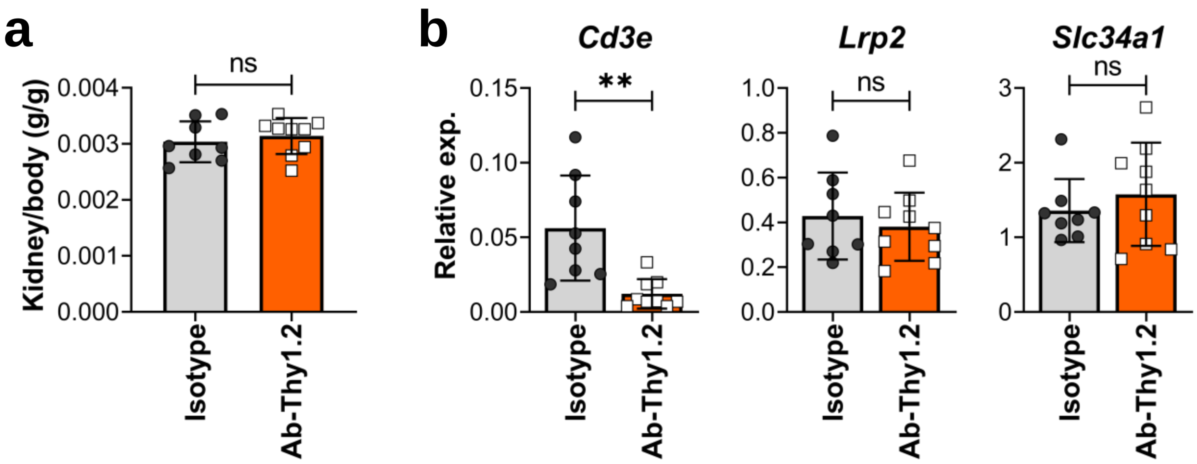

Supplementary Figure 25. Depletion of T cells alone did not prevent kidney tubule atrophy. WT mice were treated as described in Methods with either isotype or antibodies (Ab)-against Thy1.2 beginning 5 days after unilateral ischemia/reperfusion injury (U-IRI) and sacrificed on day 30. **a.** Kidney-to-body weight ratios on day 30 following U-IRI. Data are presented as mean ± SD. n=8 kidneys (isotype treatment) and n=9 kidneys (Ab-Thy1.2 treatment). ns, not statistically significant by unpaired two-tailed t test. **b.** Quantitative RT-PCR analysis for *Cd3e*, *Lrp2*, and *Slc34a1* was performed on whole kidney RNA from U-IRI mice. Data are presented as mean ± SD. n=8 kidneys (isotype treatment) and n=9 kidneys (Ab-Thy1.2 treatment). **p < 0.01; ns, not statistically significant by unpaired two-tailed t test.

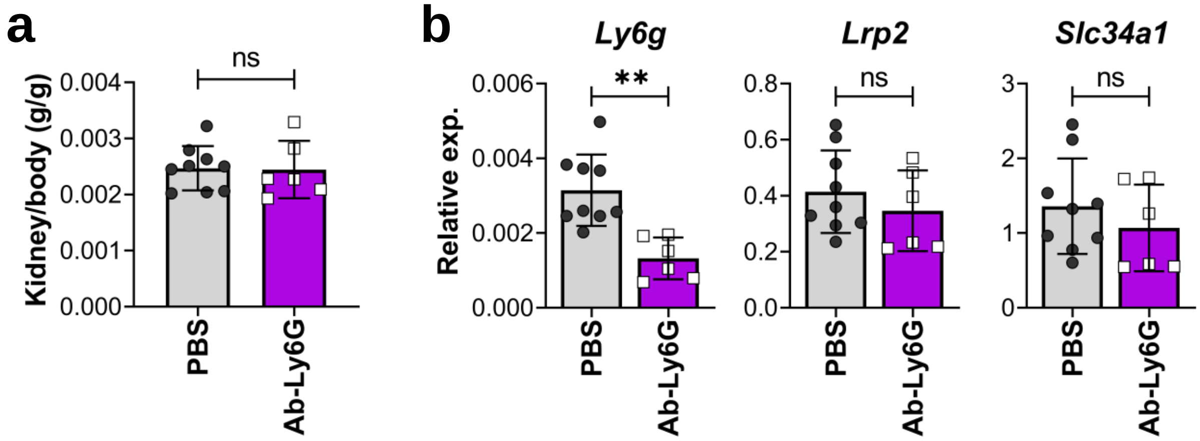

Supplementary Figure 26. Depletion of neutrophils alone did not prevent kidney tubule atrophy. WT mice were treated as described in Methods with either PBS or antibodies (Ab)-against Ly6G beginning 5 days after U-IRI and sacrificed on day 30. **a.** Kidney-to-body weight ratios on day 30 following U-IRI. Data are presented as mean ± SD. n=9 kidneys (PBS treatment) and n=6 kidneys (Ab-Ly6G treatment). ns, not statistically significant by unpaired two-tailed t test. **b.** Quantitative RT-PCR analysis for *Ly6g*, *Lrp2*, and *Slc34a1* was performed on whole kidney RNA from U-IRI mice. Data are presented as mean ± SD. n=9 kidneys (PBS treatment) and n=6 kidneys (Ab-Ly6G treatment). **$p < 0.01$; ns, not statistically significant by unpaired two-tailed t test.
