# Supplementary material for: Immune-mediated tubule atrophy promotes acute kidney injury to chronic kidney disease transition
Source: Nat Commun. 2022 Aug 19;13:4892. doi: 10.1038/s41467-022-32634-0 (PMC9391331; doi:10.1038/s41467-022-32634-0)
Supplement: Supplementary file 2 — Reporting Summary [file 41467_2022_32634_MOESM2_ESM.pdf]

nature portfolio

Corresponding author(s): Leyuan Xu and Lloyd G. Cantley

Last updated by author(s): Jul 4, 2022

Reporting Summary

Nature Portfolio wishes to improve the reproducibility of the work that we publish. This form provides structure for consistency and transparency in reporting. For further information on Nature Portfolio policies, see our Editorial Policies and the Editorial Policy Checklist.

Statistics

For all statistical analyses, confirm that the following items are present in the figure legend, table legend, main text, or Methods section.

n/a	Confirmed	
☐	☒	The exact sample size (*n*) for each experimental group/condition, given as a discrete number and unit of measurement
☐	☒	A statement on whether measurements were taken from distinct samples or whether the same sample was measured repeatedly
☐	☒	The statistical test(s) used AND whether they are one- or two-sided<br>*Only common tests should be described solely by name; describe more complex techniques in the Methods section.*
☐	☒	A description of all covariates tested
☐	☒	A description of any assumptions or corrections, such as tests of normality and adjustment for multiple comparisons
☐	☒	A full description of the statistical parameters including central tendency (e.g. means) or other basic estimates (e.g. regression coefficient) AND variation (e.g. standard deviation) or associated estimates of uncertainty (e.g. confidence intervals)
☐	☒	For null hypothesis testing, the test statistic (e.g. *F*, *t*, *r*) with confidence intervals, effect sizes, degrees of freedom and *P* value noted<br>*Give P values as exact values whenever suitable.*
☒	☐	For Bayesian analysis, information on the choice of priors and Markov chain Monte Carlo settings
☒	☐	For hierarchical and complex designs, identification of the appropriate level for tests and full reporting of outcomes
☐	☒	Estimates of effect sizes (e.g. Cohen's *d*, Pearson's *r*), indicating how they were calculated

Our web collection on statistics for biologists contains articles on many of the points above.

Software and code

Policy information about availability of computer code

Data collection	No software was used.
Data analysis	Downstream scRNA-seq data analysis was performed using the Seurat v4.0 R package. Gene set enrichment analyses were performed using the ClusterProfiler (v4.0) R package and Gene Ontology (GO) Resource. The potential ligand-receptor interaction analyses were performed using the NicheNet (v1.0) R package. The analysis code has been deposited and publicly available in Zenodo at https://zenodo.org/record/6794806#.YsL9AnaZMuU or from the authors upon request.<br>All the IHC staining slides were scanned using Aperio LV1 Real-time slide scanner, processed using Aperio ImageScope software (v12.3.2), and quantified using NIH ImageJ v1.52a. The confocal microscopy images were processed using Zeiss ZEN 2012 (blue edition).

For manuscripts utilizing custom algorithms or software that are central to the research but not yet described in published literature, software must be made available to editors and reviewers. We strongly encourage code deposition in a community repository (e.g. GitHub). See the Nature Portfolio guidelines for submitting code & software for further information.

Data

Policy information about availability of data

All manuscripts must include a data availability statement. This statement should provide the following information, where applicable:

- Accession codes, unique identifiers, or web links for publicly available datasets
- A description of any restrictions on data availability
- For clinical datasets or third party data, please ensure that the statement adheres to our policy

The scRNA-sequencing data generated in this study have been deposited and publicly available in the GEO database under accession code GSE197626.
https://www.ncbi.nlm.nih.gov/geo/query/acc.cgi?acc=GSE197626
Source data are provided with this paper. The remaining data are available within the Article or from the authors upon request.
Gene Ontology (GO) Resource database can be found at http://www.informatics.jax.org/vocab/gene_ontology/.

Human research participants

Policy information about studies involving human research participants and Sex and Gender in Research.

Reporting on sex and gender	Please see Supplementary Table 7.
Population characteristics	Please see Supplementary Table 7.
Recruitment	This is an ancillary study of a previously enrolled cohort. We prospectively enrolled participants who were scheduled to undergo a clinically indicated kidney biopsy at 2 Yale University–affiliated hospitals: Yale New Haven Hospital and St. Raphael's Hospital (both in New Haven, Connecticut, USA) from January 2015 to June 2018. We included all consecutive adult participants who met the Kidney Disease Improving Global Outcomes AKD criteria. AKD criteria include AKI and allow for a less abrupt loss of renal function over 3 months. If no baseline serum creatinine (SCr) was available to assess AKD criteria, we enrolled participants with SCr at biopsy of greater than or equal to 1.5 mg/dl. We excluded kidney transplant recipients and patients who were undergoing a kidney biopsy to evaluate a renal malignancy. We collected data through chart review of the Epic electronic health record (EHR) and cross-referenced with patient interviews.
Ethics oversight	The protocol was approved by Yale human research protection program/institutional review board.

Note that full information on the approval of the study protocol must also be provided in the manuscript.

Field-specific reporting

Please select the one below that is the best fit for your research. If you are not sure, read the appropriate sections before making your selection.

☒ Life sciences ☐ Behavioural & social sciences ☐ Ecological, evolutionary & environmental sciences

For a reference copy of the document with all sections, see nature.com/documents/nr-reporting-summary-flat.pdf

Life sciences study design

All studies must disclose on these points even when the disclosure is negative.

Sample size	The mice were sacrificed on day 1, 7, 14 and 30 after U-IRI or IRI/CL-NX (n=10 mice/end point). Baseline control mice were sacrificed and denoted as day 0 for the injury (n=10 mice) or at day 30 and designated as age-matched controls (n=9 mice). We setup the n to 10 for each model per each time point, to enhance power of this study. No sample size calculation was performed prior to the experiments.<br><br>In the scRNA-seq experiments presented in this manuscript, mice were sacrificed on day 7, 14 and 30 after U-IRI or IRI/CL-NX (n =2 kidney/model/time point). Baseline control mice (n=2 mice) were sacrificed as experiment control. No sample size calculation was performed prior to the experiment.
Data exclusions	In the scRNA-seq quality control (QC) analysis, poor quality cells with <200 unique genes and <500 unique molecular identifier (UMI) counts (likely cell fragment) and >100,000 UMI (potentially cell duplet) were excluded. Cells were excluded if their mitochondrial gene percentages were over 50%. Low-complexity cells like red blood cells with <0.8 log10 genes per UMI counts were also excluded. Only genes expressed in 5 or more cells were used for further analysis.
Replication	We have independently repeated 3 times scRNA-seq analyses and obtained the reproducible results. In our original submission, we compared the differential expressed genes and cell populations between U-IRI kidney and IRI/CL-NX kidney on day 14 after injury in the scRNA-seq experiment (n=1 mouse/model). In preparation of manuscript revision, we repeated the scRNA-seq experiment using cold-activated dissociation protocol of the mouse kidneys on day 0 (Control), day 7, and day 14 after U-IRI or IRI/CL-NX (n=2 mice/time point/model. Because of the observation of high mitochondrial contents within the samples, we then repeated the scRNA-seq experiment again using warm dissociation protocol of the mouse kidneys on day 0 (Control), day 7, day 14, and day 30 after U-IRI or IRI/CL-NX (n=2 mice/time point/model) as shown in this revised manuscript. Despite the high mitochondria content in the second experiment, all three independent experiments led

	to reproducible results. With a much higher depth of reads, we not only reproduced the data obtained from the original submission but also showed more robust analyses of cell interactions, gene set enrichment analyses, etc.
Randomization	In the mouse models of U-IRI and IRI/CL-NX, we used serum KIM-1, NGAL, creatinine, and BUN to access the initial injury. Then the mice from each model will be randomly assigned to the end point, i.e., day 1, 7, 14, and 30, to justify that the mice were subjected to the same degree of initial injury between the two models and at each time point.
Blinding	In human biopsy analyses, the investigators were blinded to group allocation during data collection and analysis. In mouse kidney histological assessment, the investigators were not blinded to group allocation during data collection and analysis because the phenotypes of the kidneys are markedly different between U-IRI and IRI/CL-NX (atrophy vs hypertrophy).

Reporting for specific materials, systems and methods

We require information from authors about some types of materials, experimental systems and methods used in many studies. Here, indicate whether each material, system or method listed is relevant to your study. If you are not sure if a list item applies to your research, read the appropriate section before selecting a response.

Materials & experimental systems

n/a	Involved in the study
☐	☒ Antibodies
☒	☐ Eukaryotic cell lines
☒	☐ Palaeontology and archaeology
☐	☒ Animals and other organisms
☐	☒ Clinical data
☒	☐ Dual use research of concern

Methods

n/a	Involved in the study
☒	☐ ChIP-seq
☒	☐ Flow cytometry
☒	☐ MRI-based neuroimaging

Antibodies

Antibodies used	Primary rabbit monoclonal antibodies against F4/80 (#70076, Cell Signaling Technology, 1:100 dilution), CD11c (#97585, Cell Signaling Technology, 1:100 dilution), Ly6G (#87048S, Cell Signaling Technology, 1:100 dilution), CD3ε (#99940, Cell Signaling Technology, 1:100 dilution), CD4 (#25229, Cell Signaling Technology, 1:100 dilution), CD8α (#98941, Cell Signaling Technology, 1:100 dilution), GAPDH (HRP conjugate) (#3683, clone:14C10, Cell Signaling Technology, 1:1000 dilution).<br>Primary rabbit polyclonal antibody against mouse megalin [anti-MC220 (PMID: 15180987), original obtained from Daniel Biemesderfer at the Yale Nephrology, 1:100 dilution]<br>Primary mouse monoclonal antibody against KSP-Cadherin [clone:4F6/F6, PMID: 9721215, kindly provided by Robert Brent Thomson at the Yale Nephrology, 1:100 dilution]<br>Primary rabbit polyclonal antibody against UMOD (#sc-20631, clone:H-135, Santa Cruz Biotechnology, 1:100 dilution)<br>Primary goat polyclonal antibody against TIM-1/KIM-1/HAVCR (#AF1817, Novus Biologicals, 1:100 dilution)<br>Primary rat monoclonal antibody against F4/80 (#MCA497, Clone:A3-1, Bio-Rad, 1:100 dilution)<br>Primary mouse monoclonal antibody against CD3ε (#NBP2-53387, clone:C3e/1308, Novus Biologicals, 1:100 dilution)<br>Primary mouse monoclonal antibody against CD66b (#305102, clone:G10F5, BioLegend, 1:100 dilution)<br>Primary rabbit polyclonal antibody against megalin (#19700-1-AP, Thermo Fisher Scientific, 1:100 dilution)<br>Biotinylated LTL (#B-1325, Vector Laboratories, 1:200 dilution)
Validation	All primary antibodies were commercially purchased (datasheets are available on their websites) except antibodies against megalin (anti-MC220) and KSPCadherin (clone:4F6/F6). These two antibodies have been validated and published in previous publications (PMID: 15180987 and PMID: 9721215, respectively).

Animals and other research organisms

Policy information about studies involving animals; ARRIVE guidelines recommended for reporting animal research, and Sex and Gender in Research

Laboratory animals	C57BL/6 (Envigo) wild-type mice (age 9-11 weeks) were used in this work. All mice were maintained on a 12-hour light and 12-hour dark cycle at the temperature between 68-79 °F and humidity between 30-70% with free access to standard food and water before and after surgery.
Wild animals	This work does not involve any wild animals.
Reporting on sex	Due to the substantial difference in susceptibility to IRI injury between male and female mice (PMID: 22993069), male mice were exclusively used to reduce total numbers of mice required for statistical analysis.
Field-collected samples	No field-collected samples were used in the study as the information is not relevant to this work.
Ethics oversight	All animal protocols were approved by the Yale University Animal Care and Use Committee (IACUC protocol number 10538).

Note that full information on the approval of the study protocol must also be provided in the manuscript.

Clinical data

Policy information about clinical studies

All manuscripts should comply with the ICMJE guidelines for publication of clinical research and a completed CONSORT checklist must be included with all submissions.

Clinical trial registration	*Provide the trial registration number from ClinicalTrials.gov or an equivalent agency.*
Study protocol	*Note where the full trial protocol can be accessed OR if not available, explain why.*
Data collection	*Describe the settings and locales of data collection, noting the time periods of recruitment and data collection.*
Outcomes	*Describe how you pre-defined primary and secondary outcome measures and how you assessed these measures.*
